# Computational-guided discovery of UDP-glycosyltransferases for lauryl glucoside production using engineered *E. coli*

**DOI:** 10.1186/s40643-024-00820-1

**Published:** 2024-10-26

**Authors:** Kasimaporn Promubon, Kritsada Tathiya, Aussara Panya, Wasu Pathom-Aree, Pachara Sattayawat

**Affiliations:** 1 Department of Biology, Faculty of Science, Chiang Mai University, Chiang Mai, 50200 Thailand; 2 Cell Engineering for Cancer Therapy Research Group, Faculty of Science, Chiang Mai University, Chiang Mai, 50200 Thailand; 3 Master of Science Program in Applied Microbiology (International Program), Faculty of Science, Chiang Mai University, Chiang Mai, 50200 Thailand

**Keywords:** UDP-glycosyltransferase, Lauryl glucoside, Computational method, 1-Dodecanol, Engineered *E. coli*, Novel biosynthetic pathway, Genome mining

## Abstract

Defining suitable enzymes for reaction steps in novel synthetic pathways is crucial for developing microbial cell factories for non-natural products. Here, we developed a computational workflow to identify C12 alcohol-active UDP-glycosyltransferases. The workflow involved three steps: (1) assembling initial candidates of putative UDP-glycosyltransferases, (2) refining selection by examining conserved regions, and (3) 3D structure prediction and molecular docking. Genomic sequences from *Candida*, *Pichia*, *Rhizopus*, and *Thermotoga*, known for lauryl glucoside synthesis via whole-cell biocatalysis, were screened. Out of 240 predicted glycosyltransferases, 8 candidates annotated as glycosyltransferases were selected after filtering out those with signal peptides and identifying conserved UDP-glycosyltransferase regions. These proteins underwent 3D structure prediction and molecular docking with 1-dodecanol. RO3G, a candidate from *Rhizopus delemar* RA 99–880 with a relatively high ChemPLP fitness score, was selected and expressed in *Escherichia coli* BL21 (DE3). It was further characterized using a feeding experiment with 1-dodecanol. Results confirmed that the RO3G-expressing strain could convert 1-dodecanol to lauryl glucoside, as quantified by HPLC and identified by targeted LC-MS. Monitoring the growth and fermentation profiles of the engineered strain revealed that RO3G expression did not affect cell growth. Interestingly, acetate, a major fermentation product, was reduced in the RO3G-expressing strain compared to the GFP-expressing strain, suggesting a redirection of flux from acetate to other pathways. Overall, this work presents a successful workflow for discovering UDP-glycosyltransferase enzymes with confirmed activity toward 1-dodecanol for lauryl glucoside production.

**Graphical abstract:**

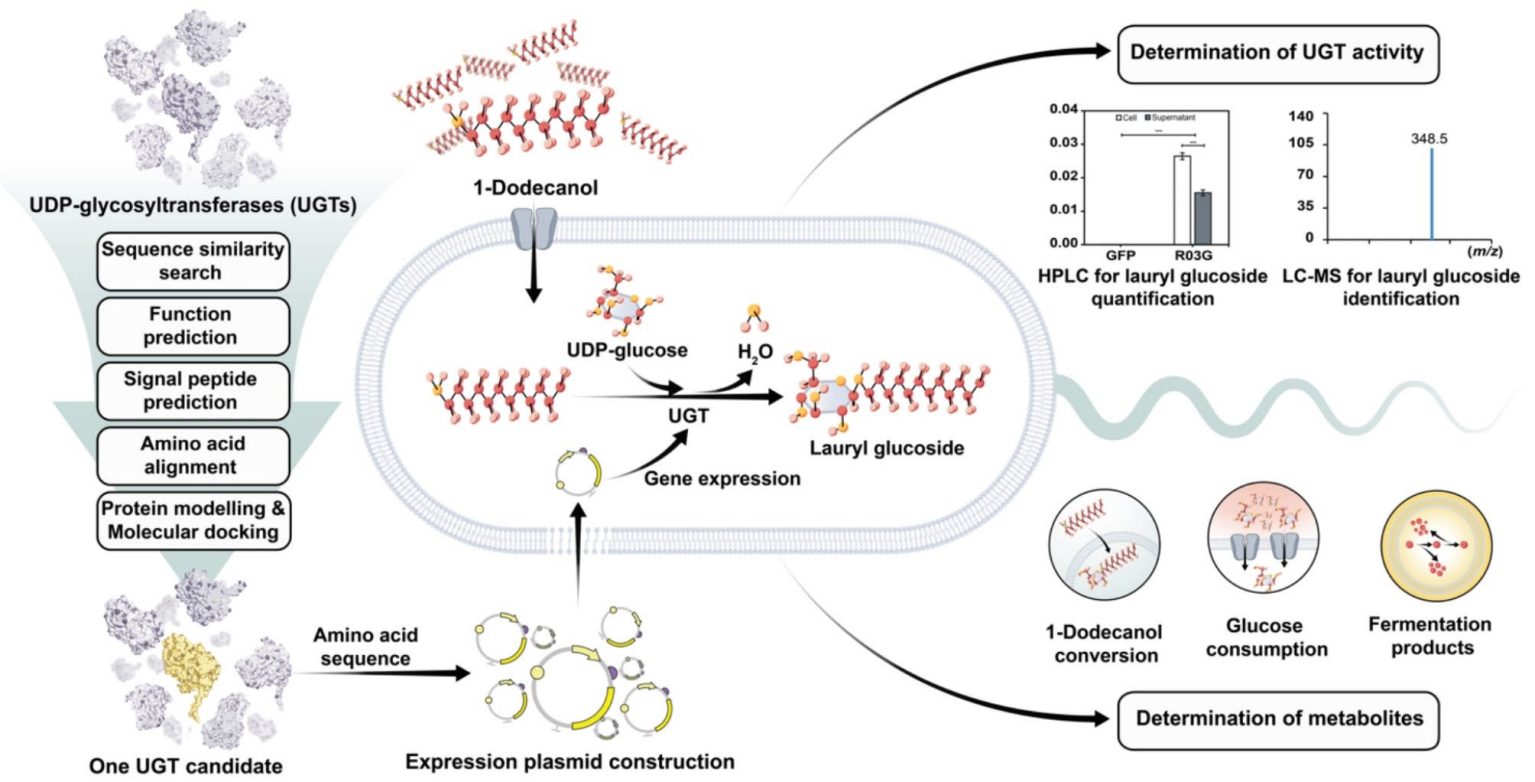

**Supplementary Information:**

The online version contains supplementary material available at 10.1186/s40643-024-00820-1.

## Introduction

Bio-based production is one of the alternatives for producing attractive chemicals, as it utilizes microbial cells as factories and benefits from their native ability to synthesize the compounds of interest. With a little help from genetic modification, novel synthetic pathways can also be implemented inside the microbial cells. Several bio-based chemicals are commercially available (Cho et al. 2022), which further emphasizes the feasibility of microbial cell factories. Lauryl glucoside (also known as dodecyl β-D-glucopyranoside or dodecanoyl glucoside) is a commercially appealing compound that is used as a non-ionic surfactant in a variety of cosmetic products. It is biodegradable and low in toxicity, which makes it even more commercially attractive (Fiume et al. 2013). This chemical is currently produced from a condensation of a straight-chain alcohol and a cyclic form of glucose (*D*-glucopyranose) (Fiume et al. 2013), which is considered unsustainable because these substrates are either derived from petroleum-based or plant-based chemicals (Sorrell et al. 2010).

Due to its high value, this work aims to overcome the challenges of current lauryl glucoside production by utilizing microbial cell factories. However, there are no known organisms or biosynthetic pathways that can naturally synthesize this compound. Previously, octyl glucoside production from an engineered *E. coli* was reported using a novel synthetic pathway that included *O*-glycosylation of 1-octanol as a final step (Sattayawat et al. 2020). Based on this work, we hypothesized that it would be feasible to implement a similar metabolic pathway using 1-dodecanol as one of the substrates for the final reaction step given that 1-dodecanol production has been achieved through a few synthetic pathways in *E. coli* and cyanobacteria (Hsieh et al. 2018; Yunus and Jones 2018). However, the enzyme responsible for the final reaction, *O*-glycosylation, has yet to be identified. UDP-glycosyltransferases are the primary choice as it has been used in the aforementioned work in *E. coli* for octyl glucoside production (Sattayawat et al. 2020). Yet, preliminary searches have shown that UDP-glycosyltransferases with activity toward 1-dodecanol have not been reported. Moreover, to optimize pathway flux, enzymes with high specificity toward the substrate of interest are preferred. Furthermore, no organisms that naturally synthesize lauryl glucoside from their metabolite substrates, have been identified indicating the incomplete synthetic pathways for this compound. However, this does not imply a lack of enzymes capable of glycosylating 1-dodecanol. Therefore, organisms capable of catalyzing the conversion of 1-dodecanol to its corresponding glucoside were further investigated. *Candida molischiana* 35M5N, *Pichia etchellsii* and *Thermotoga neapolitana* were reported to possess glycoside hydrolase activity for lauryl glucoside synthesis (Gueguen et al. 1995; Pozzo et al. 2010; Younis Rather et al. 2012) whereas the glycosyltransferase activity was proposed in *Rhizopus stolonifera* NRRL 1478 for lauryl glucoside synthesis (El-Sharkawy 1996).

In this work, computational-guided methods were used for the search of putative UDP-glycosyltransferases with activity toward 1-dodecanol for lauryl glucoside production in *E. coli*. With the aforementioned information, genomes of 4 organisms; *Candida*, *Pichia*, *Rhizopus* and *Thermotoga*, were screened using model UDP-glycosyltransferases from *Arabidopsis thaliana* as referenced templates. To narrow down the number of matches, function prediction, subcellular localization prediction and conserved region identification were performed, and 8 potential putative sequences were obtained. All potential candidates were then subjected to Colabfold structure modelling and molecular docking to predict the 3D structures and computationally confirm the binding of candidates with 1-dodecanol. One putative enzyme from *R. delemar* RA 99–880, namely RO3G, showed the most potential. RO3G was then expressed and characterized in *E. coli* BL21 (DE3). The synthesis of lauryl glucoside using RO3G was investigated through a 1-dodecanol feeding experiment. Targeted LC-MS analysis revealed the production of lauryl glucoside by engineered *E. coli* BL21 (DE3) harboring pCDF-RO3G.

## Materials and methods

### Computational-guided discovery of UDP-glycosyltransferases

**Fig. 1:**
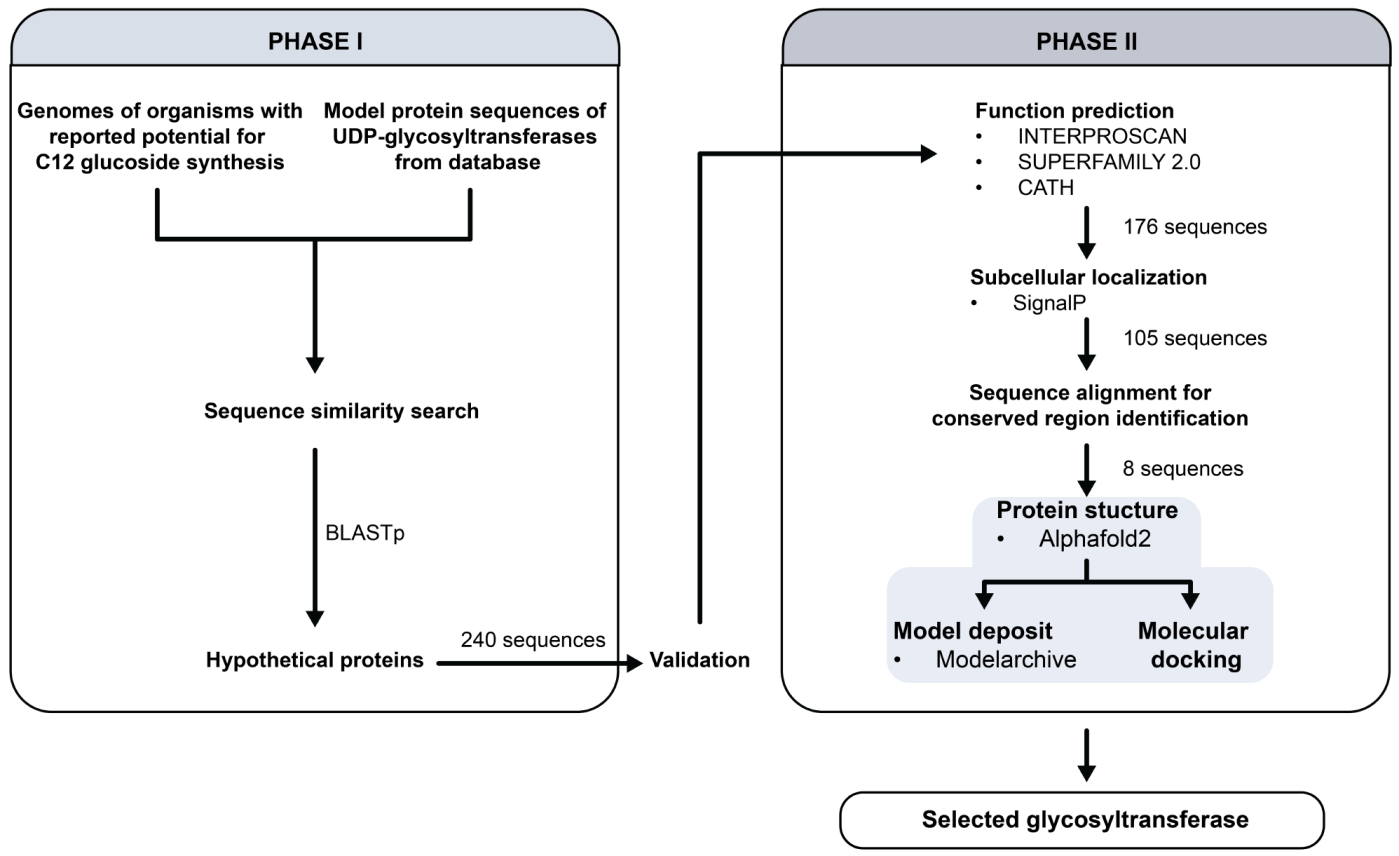
A computational workflow for putative UDP-glycosyltransferase discovery for lauryl glucoside production. Phase I is the discovery of hypothetical proteins with potential to be glycosyltransferases and Phase II is the computational characterization of the hypothetical proteins

In this work, a computational workflow for UDP-glycosyltransferase discovery was developed and presented in Fig. 1. The URLs of all tools and databases used in this work are listed in Supplementary Table 1.

### Sequence similarity search

As glycosyltransferases are a large group of enzymes with many reported proteins, in this work, the sequences of glycosyltransferases exported from the *Arabidopsis* Glycosyltransferase Family 1 database (http://www.p450.kvl.dk/UGT.shtml#seqs) were used as templates. *Arabidopsis* is known to possess more than 100 genes encoding known or likely UDP-glycosyltransferases that in many cases are well-characterized (Ross et al. 2001; Gharabli et al. 2023). Moreover, the use of *Arabidopsis* UDP-glycosyltransferases as templates for similar purposes has been demonstrated. For example, all UDP-glycosyltransferase sequences from *Arabidopsis* was used to mine for UDP-glycosyltransferases from *Lotus japonicus* (Yin et al. 2017; Pei et al. 2022). All sequences can be downloaded from the web-based database, which further facilitates the workflow. All UDP-glycosyltransferase sequences were exported as FASTA files using the function “Arabidopsis UGT protein sequence file”. A total of 122 protein sequences were subjected to BLASTp against the genomes of potential organisms with confirmed activity for lauryl glucoside synthesis listed in Table 1. Non-redundant protein sequences (nr) database and blastp (protein-protein BLAST) algorithm were selected for this step to ensure comprehensive, efficient, and accurate identification of homologous proteins. Once the search showed results, only hypothetical protein sequences with an E-value of less than 1 × 10^− 10^ were selected.

**Table 1:** Reported organisms with lauryl glucoside synthesis capability with their available genomes in NCBI database

Reported organism	Available genome in NCBI database	Remark	Reference
*Candida molischiana* 35M5N	*Candida* (taxid:5475)	Reported enzymatic synthesis of lauryl glucoside	Gueguen et al. 1995
*Pichia etchellsii*	*Pichia* (taxid:4919)	Reported synthesis of lauryl glucoside	Younis Rather et al. 2012
*Rhizopus stolonifera* NRRL 1478	*Rhizopus* (taxid:4842)	Reported transformation of lipids to lauryl glucoside	El-Sharkawy 1996
*Thermotoga neapolitana*	*Thermotoga* (taxid:28240)	Reported enzymatic synthesis of lauryl glucoside	Pozzo et al. 2010

### Sequence-based function prediction

To computationally predict the functions of the retrieved protein sequences, three different webservers; InterProScan, SUPERFAMILY 2.0 and CATH (Supplementary Table 1) were selected. All these webservers were previously used to annotate hypothetical proteins from whole genome sequences and shown to be relatively comparable (Uttarotai et al. 2022). As in this work, many matches were retrieved from the sequence similarity search, therefore, sequences predicted to be glycosyltransferases with at least two out of three webservers were selected for further steps.

### Subcellular localization

SignalP was used to investigate the subcellular localization of the putative glycosyltransferases. It should be noted that glycosyltransferases are enzymes that could be either membrane-bound or soluble in cell cytoplasm, yet the glycosyltransferases with no signal peptides were to be selected to facilitate the downstream engineering processes in *E. coli*. In this work, SignalP 6.0 (Supplementary Table 1) was used as it is the most developed version released in 2022 (Teufel et al. 2022). The cut off value was 0.99. The putative protein sequences that do not contain signal peptides according to SignalP prediction were selected for the following steps.

### Sequence alignment

Amino acid sequences of the candidates were aligned using Clustal Omega (Supplementary Table 1). The UGT conserved region, WAPQVAILHHPSTQLFLTHGGAGSVYEALYKGVPIVVYPFFGFQ, (Xie et al. 2017) and the sequences involved in catalytic activity specific for fungi (Taujale et al. 2020), DVD and xET, were identified. The presence of these regions was used to narrow down the number of candidates for further steps.

### Protein structure prediction and validation

Alphafold2 on the Google Colab platform (Supplementary Table 1) was used to model the 3D structures of the narrowed-down candidates using default parameters as follows. The num_relax was 1, the template mode was none. Msa_mode and pair_mode were mmseqs2_uniref_env and unpaired_paired, respectively. The model_type, num_recycles and recycle_early_stop_tolerance were all set as auto. Pairing strategy was greedy. Max_msa was auto and num_seeds was 1. All protein structures were validated using Ramachandran plot via Procheck online (Supplementary Table 1). The acceptable protein structures with over 90% in the most favored regions were considered high-quality models according to the MolProbity guidelines and were subjected further to molecular docking analysis. All predicted protein structures were deposited in ModelArchive database for public use.

### Molecular docking

As to further decrease the number of putative enzyme candidates, molecular docking was performed using the 3D models of the candidates from the previous step and 1-dodecanol as ligands. The chemical structure of 1-dodecanol was obtained from PubChem databank (Supplementary Table 1). Molecular docking was performed using GOLD Protein–ligand docking software (Verdonk et al. 2003). The fitness score from molecular docking was considered and the candidate was used further. The best docking poses were further analyzed using Discovery Studio Visualizer (BIOVIA 2017). To identify the binding pocket of all newly discovered candidates, the binding pocket of a well-characterized UDP-glycosyltransferase from *A. thaliana*, UGT72B1, and its ligand were used as references.

## In vivo characterization of the computational characterized UDP-glycosyltransferase

### Expression of the selected UDP-glycosyltransferase and lauryl glucoside analysis

One candidate of the putative glycosyltransferases was selected and synthesized as a gene and transformed into *E. coli* BL21(DE3) as an expression host. A commercial vector, pCDFDuet-1, was used as an expression system. Codon optimization for *E. coli* expression was also performed using the service from the manufacturer (Beijing Tsingke Biotech Co., Ltd., China). The gene sequence is presented in Supplementary Data 1. A plasmid with pCDF-backbone carrying Green Fluorescent Protein (GFP) gene was used as a control. The strains expressing UDP-glycosyltransferase or GFP were cultivated in M9 minimal media with 2% (w/v) glucose and induced with 0.5 mM isopropyl β-D-1-thiogalactopyranoside (IPTG) at the beginning of the incubation. The culture was then incubated at 30 °C in a shaking incubator at 150 rpm. After 2 h, the culture was fed with 2.0 mM 1-dodecanol (Sigma-Aldrich, USA) and continuously incubated under the same conditions for a total of 48 h. Thereafter, chloroform:methanol (2:1) was used for the extraction of lauryl glucoside. To quantify lauryl glucoside, an Agilent Technologies 1260 Infinity II high-performance liquid chromatography (HPLC) system was used with a corresponding lauryl glucoside standard (Biosynth, Switzerland). A 50 µL sample was injected into the HPLC, equipped with an EC-C-18 column (Agilent InfinityLab Poroshell 120, 4.6 × 150 mm, 4-micron) and a refractive index (RI) detector (Agilent G1362A). The sample was separated and eluted from the column using acetonitrile:water (75:25) at a flow rate of 0.8 mL/min, with the column temperature set at 45 °C (Younis Rather et al. 2012). Lauryl glucoside in the extracted samples was also identified using targeted Liquid Chromatography - Mass Spectrometry (LC-MS), corresponding to the lauryl glucoside standard. A 10 µL sample was injected into an Agilent 6130 Single Quadrupole LC-MS system equipped with a C-18 column (RESTEK, 4.6 × 150 mm, 4-micron). The sample passed through the column using a mobile phase of 60:40 acetonitrile:water at a flow rate of 0.6 mL/min, with the column temperature set at 30 °C. Mass spectrometry was performed in ESI source negative mode, targeting *m/z* 348.48. The ion source temperature was set at 300 °C, the capillary voltage at 3.5 kV, and the fragmentor at 135 V. The nebulizer gas pressure was set at 45 psi (Xue et al. 2021).

### 1-Dodecanol analysis

During cultivation, samples were collected for the analysis of 1-dodecanol. Five mL of cultures were collected at 48 h and extracted with 10% (v/v) dodecane. The extracted samples were then analyzed using Gas Chromatography-Flame Ionization Detector (GC-FID). An Agilent 7890B GC, 7693 autosampler and G4513A injector system equipped with HP-5 column (Agilent 1909J1-419, 3 m × 320 μm × 0.25 μm, 7 inch) and a FID was used to detect and quantify 1-dodecanol corresponding to a 1-dodecanol (Sigma-Aldrich, USA) standard. One µL of samples was directly injected into the system, with a split ratio of 100:1. After injection, the sample was flamed at 250 °C and passed into a column in an oven, where the initial temperature was set at 40 °C, held for 3 min before increasing up to a final temperature of 250 °C at 35 °C/min. Helium, at a flow rate of 5.6 mL/min, was used as the carrier gas. The detector operated at a temperature of 250 °C. The gases used in the system were air (400 mL/min), hydrogen gas (30 mL/min), and nitrogen gas (25 mL/min) all at a temperature of 250 °C (Pulungan et al. 2018).

### Glucose and fermentation product analysis

To monitor the cell growth, 1 mL of culture was sampled every 24 h and the optical density was measured using spectrophotometry at 600 nm, along with glucose consumption analysis by HPLC. An additional 1 mL of culture was also collected for fermentation product analysis. Ethanol was quantified using GC-FID as described in the previous section corresponding to an ethanol (RCI labscan, Thailand) standard, while acetate, formate, and lactate were analyzed using HPLC. An Agilent Technologies 1260 Infinity II High performance liquid chromatography (HPLC) system was used to detect and quantify glucose, and fermentation products, including acetate, formate, and lactate, by comparing the retention times and area under the curve with *D*-glucose (VWR, USA), acetate (RCI labscan, Thailand), formate (Fischer, USA), and lactate (Lobachemie, India). For glucose, a 10 µL sample was injected into the HPLC equipped with NH2P-50 4E column (Shodex Asahipak 4.6 × 250 mm 5-micron) and an RI detector (Agilent G1362A). The sample were separated and eluted from the column by acetonitrile:water (75:25) with a flow rate of 0.8 mL/min, and the column temperature was set at 30 °C. For fermentation product analysis, the HPLC equipped with Inertsil ODS-3 C-18 column (GL Science, 4.6 × 150 mm 5-micron) and a UV detector set at 210 nm (Agilent G7115A) was used. Samples (50 µL) were injected into the system and eluted from the column with 20 mM KH_2_PO_4_ elution buffer at pH 2.4, at a flow rate of 0.8 mL/min, with the column temperature set at 25 °C (Kaewkod et al. 2019).

### Statistical analysis

To determine significant differences between treatments, a two-sided Student’s *t*-test was used. The *p*-value ≤ 0.05 was considered significantly different.

## Results

### Homology-based screening of UDP-glycosyltransferases

#### Sequence similarity search

The protein sequences of UDP-glycosyltransferases (UGTs) from *Arabidopsis thaliana* were retrieved from *Arabidopsis* Glycosyltransferase Family 1 database. All protein sequences were exported and searched against the genomes of the reported genera (Table 2) with potential of having C12-active UGTs. Interestingly, it was found that when the “Quick BLASTp” function was selected, none of the 122 protein sequences from the *Arabidopsis* database showed any matches. However, using the “BLASTp” function resulted in a total of 240 matches. The number of matched sequences for each genome were different as shown in Table 2.

**Table 2:** Results from BLASTp search against the genomes of reported organisms

Genome	Number of matched protein sequences*
*Candida* (taxid:5475)	43
*Pichia* (taxid:4919)	8
*Rhizopus* (taxid:4842)	189
*Thermotoga* (taxid:28240)	0
**Total**	**240**

*Only matches with E-value of ≤ 1 × 10^− 10^ were selected

#### Sequence-based function prediction

The function of all 240 matched sequences was predicted using different webservers, InterProScan, SUPERFAMILY 2.0 and CATH. Since some of the matched proteins had previously been reported as glycosyltransferases, and the primary goal of this work was to discover uncharacterized enzymes, only the hypothetical proteins were considered. Of 240 sequences, 176 were selected for further studies, with at least two out of three webservers predicting them to be glycosyltransferases. All the annotation results are presented in Supplementary Table 2.

#### Subcellular localization

**Fig. 2:**
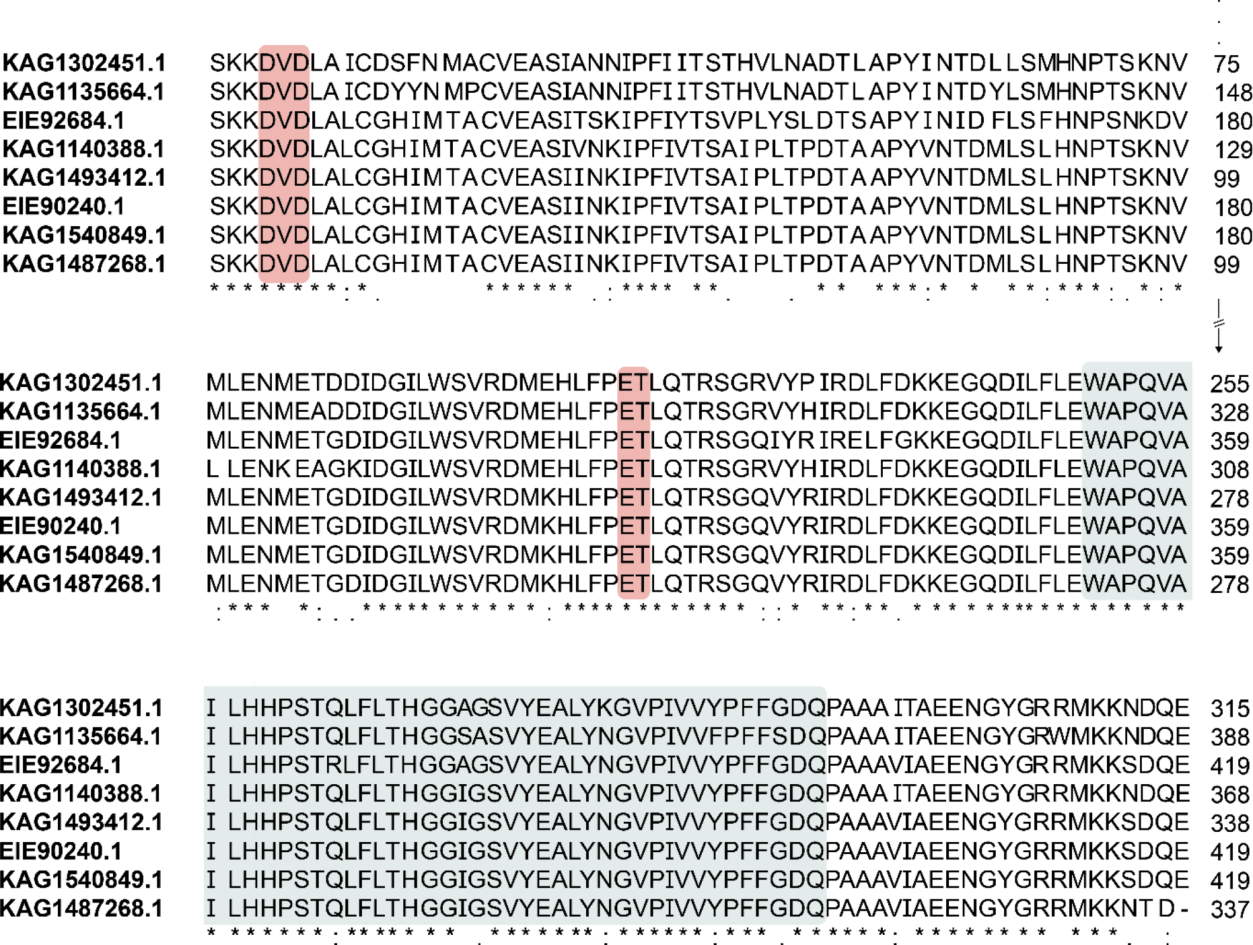
Amino acid sequence alignment of 8 candidates from *Rhizopus*. The UGT conserved motif is indicated in the blue box and catalytic motifs specific for *Rhizopus* glycosyltransferase (GT62) are indicated in the red box

Signal peptides of all proteins were predicted using SignalP 6.0 and only the candidates with no predicted signal peptides were selected. Theoretically, subcellular localization of glycosyltransferases can either be in cytoplasm or bound to cell membrane. However, in this work, UDP-glycosyltransferase is expected to be expressed in the cytoplasm to catalyze 1-dodecanol synthetized in the cytoplasm. Moreover, from a heterologous protein expression perspective, it is known that the expression of cell membrane-binding proteins could lead to the formation of inclusion bodies (Bhatwa et al. 2021). Thus, only the proteins without predicted signal peptides are of interest. A total of 105 protein sequences were further investigated. Since this number remains high, all candidates were further narrowed down to 40 proteins predicted as “UDP- glycosyltransferase” from all webservers. These 40 candidates were then subjected to an amino acid sequence alignment. The alignment allowed visualization of whether the proteins contain appropriate conserved regions or not. A UGT conserved region and catalytic conserved regions were identified. In the case of GT62 – a group of *Rhizopus* glycosyltransferases – the catalytic conserved regions are DVD and xET residues (Taujale et al. 2020). The results revealed 8 UDP-glycosyltransferases that fit into this definition, as depicted in Fig. 2.

#### Protein structure prediction and validation

All 8 protein sequences were successfully modeled using Alphafold2 via ColabFold. All structures were then validated using Ramachandran plots and the validated protein structures were deposited in ModelArchive (Table 3) for public use. All modeled structures exhibited average pLDDT scores higher than 90, indicating high confidence in the models. According to the Ramachandran plots, residues with values in the most favored regions above 90% were considered acceptable. In this study, all modeled structures met this criterion. All candidates and related information are listed in Table 3.

**Table 3:** A list of shortlisted candidates and the related information

Candidate (Accession no.)	Original host	Average pLDDT scores	Procheck - Residues in most favored regions	ModelArchive ID
EIE90240.1	*Rhizopus delemar* RA 99–880	92.8	93.9	ma-c4qpa
KAG1140388.1	*Rhizopus arrhizus*	91.9	94.1	ma-kngby
KAG1302451.1	*Rhizopus arrhizus*	93.2	92.6	ma-5bthn
KAG1487268.1	*Rhizopus delemar*	91.6	94.6	ma-6oeo7
KAG1493412.1	*Rhizopus delemar*	92.5	94.7	ma-plq92
KAG1540849.1	*Rhizopus delemar*	93.1	93.2	ma-ros8a
KAG1135664.1	*Rhizopus arrhizus*	91.6	92.9	ma-z7t38
EIE92684.1	*Rhizopus delemar* RA 99–880	92.7	93.9	ma-d29mm

#### Molecular docking of modeled proteins with 1-dodecanol

**Fig. 3:**
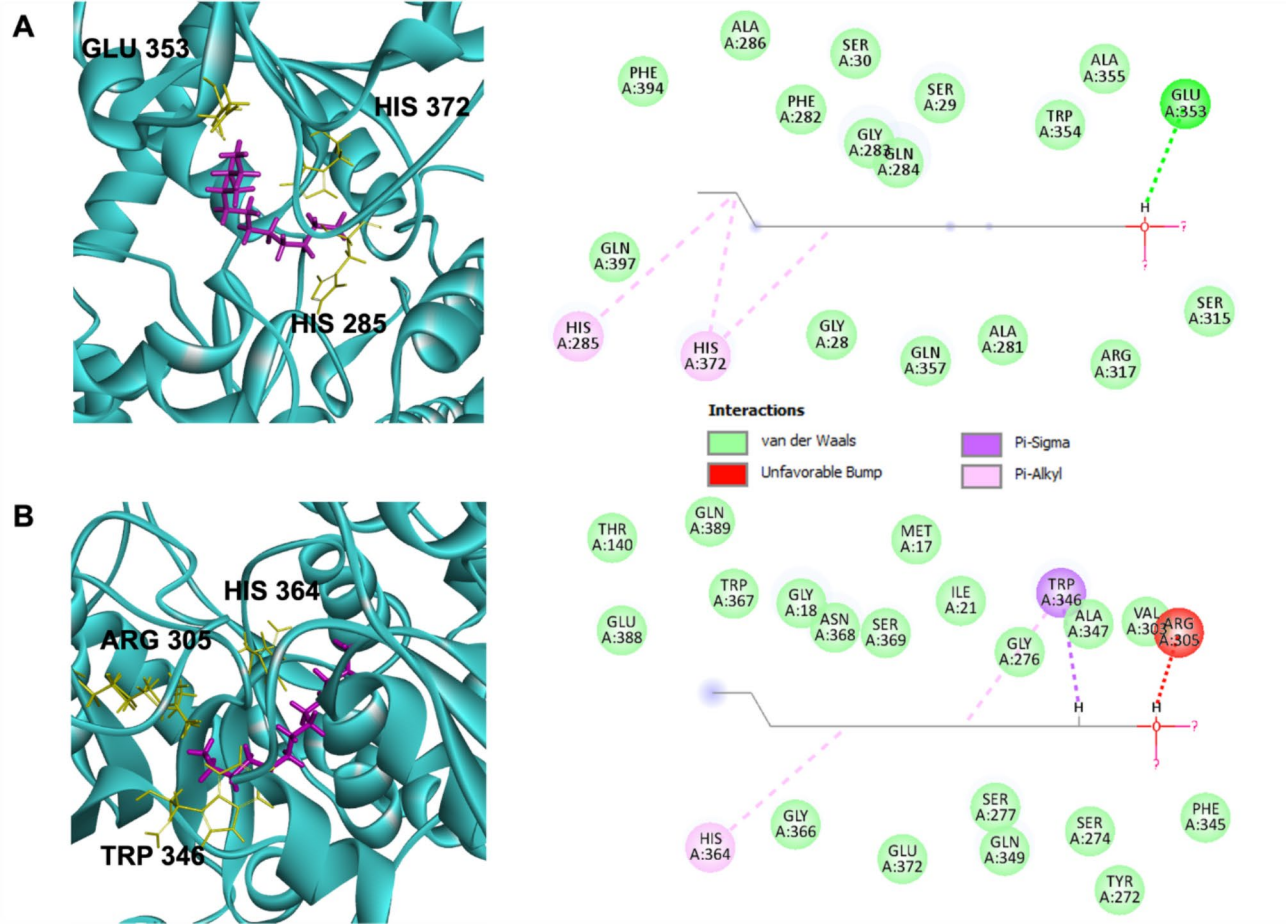
Molecular docking of a UDP-glycosyltransferase candidate and 1-dodecanol. Binding poses of (**A**) the most promising putative UDP-glycosyltransferase (Accession no. EIE92684.1) with 1-dodecanol, compared to (**B**) UGT72B1 (Accession no. OAP00532.1)

To perform molecular docking, the binding pocket of each enzyme candidate needs to be identified. A UDP-glycosyltransferase from *A. thaliana*, UGT72B1, was used as a reference for binding pocket identification. The fitness scores resulted from GOLD docking using ChemPLP fitness score are presented in Supplementary Table 3. As demonstrated, all candidates showed similar fitness scores ranging between 44.78 and 50.66 with the highest values from KAG1540849.1; however, this candidate was retrieved from *R. delemar*, which was considered a pathogenic species as well as a more clinical severe *R. arrhizus.* This left *R. delemar* RA 99–880, a well-characterized strain with reported whole genome sequence (Ma et al. 2009) and genes encoding enzymes that have been successfully cloned and expressed in *E. coli* (Xiao et al. 2014) as a potential source. A candidate with accession no. EIE90240.1, which showed the highest ChemPLP fitness score among the candidates from *R. delemar* RA 99–880, has previously been reported for its UDP-glycosyltransferase activity (Xie et al. 2017). Since the aim of this study was to discover novel UDP-glycosyltransferases, we selected the RO3G_17395 gene (Accession no. EIE92684.1), which had the second-highest ChemPLP fitness score, for further in vivo characterization. The structure of the potential UDP-glycosyltransferase candidate (Accession no. EIE92684.1) with its substrate binding pocket are presented in Fig. 3 in comparison with the reference UDP-glycosyltransferase (UGT72B1). Amino acid residues responsible for the interactions between RO3G and 1-dodecanol are ILE285, ALA355 and HIS372 whereas they are ARG306, TRP346 and HIS364 in the case of reference. As shown, for both proteins, histidine is the interactive amino acid residue that interacts with 1-dodecanol through a pi-alkyl bond.

### Heterologous expression and characterization of the novel UDP-glycosyltransferase

**Fig. 4:**
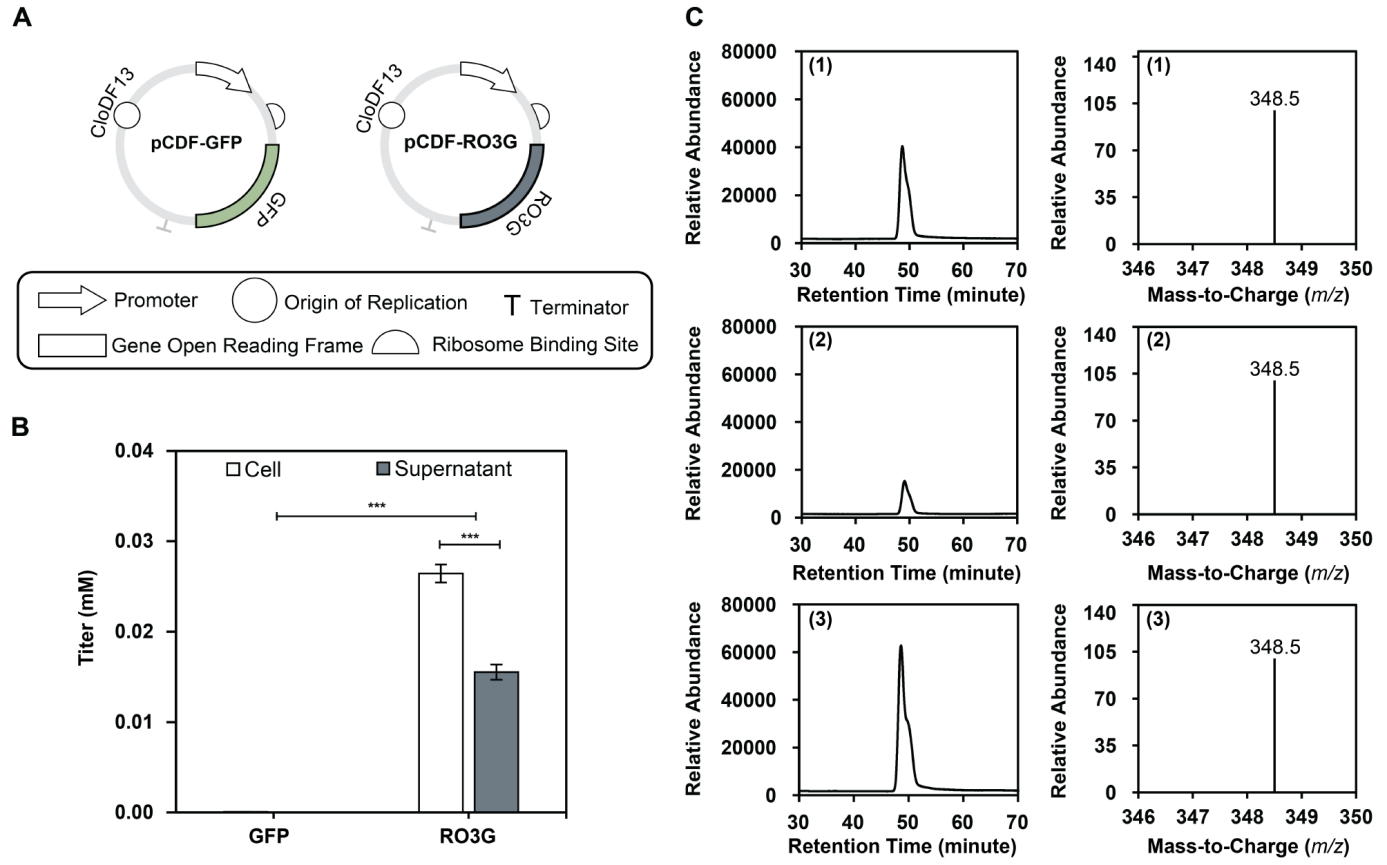
Lauryl glucoside synthesis from engineered *E. coli* BL21 (DE3) using the newly discovered UDP-glycosyltransferase, RO3G. (**A**) Maps of pCDF-based plasmids harboring GFP and RO3G genes, (**B**) Synthesis of lauryl glucoside quantified using HPLC and (**C**) LC-MS chromatograms of (1) lauryl glucoside standard (2) GFP (3) RO3G samples. *E. coli* BL21 (DE3) harboring pCDF-GFP or pCDF-RO3G were cultivated in M9 minimal medium with 2% (w/v) glucose fed with 2.0 mM 1-dodecanol. Asterisk (*) indicates *p*-values: * *p* ≤ 0.05, ** *p* ≤ 0.01 and *** *p* ≤ 0.005

The UDP-glycosyltransferase candidate (Accession no. EIE92684.1), derived from the RO3G_17395 gene of *R. delemar* RA 99–880 and hereafter referred to as RO3G, was further characterized. The RO3G gene was synthesized in a pCDF-based plasmid and transformed into *E. coli* BL21 (DE3) in comparison with a control strain harboring a pCDF-GFP plasmid (Fig. 4A). In this work, a plasmid containing GFP was used as a control because GFP does not affect cellular metabolism, thereby allowing us to investigate the metabolic effects of the UDP-glycosyltransferase expression. This choice helps to ensure that any observed changes can be attributed specifically to the heterologous protein rather than to the plasmid burden alone. Lauryl glucoside synthesis was tested using M9 minimal media with 2% (w/v) glucose in a 1-dodecanol feeding experiment. The engineered strain successfully converted 1-dodecanol to lauryl glucoside, as quantified by HPLC. Lauryl glucoside was detected in both cell and supernatant fractions (Fig. 4B). Although the total titer of lauryl glucoside was low (0.026 ± 0.001 mM in the supernatant and 0.016 ± 0.001 mM in the cell pellet) given the 2.0 mM 1-dodecanol feed. The samples were also identified using LC-MS ESI-MS in the negative ionization mode. Both the retention time and mass spectrum were compared with a lauryl glucoside standard. The sample’s peak appeared at approximately 48 min of retention time with a molecular mass of ([M-H]- *m/z* = 348.5), matching the lauryl glucoside standard. This indicated that the UDP-glycosyltransferase, RO3G, functioned in *E. coli* BL21 (DE3) and transfer the sugar moiety from UDP-glucose to 1-dodecanol, thereby synthesizing lauryl glucoside. However, it should be noted that a small peak was observed in the *E. coli* BL21 (DE3) harboring pCDF-GFP without the additional heterologous expression of UDP-glycosyltransferase, suggesting the presence of a native glycosyltransferase in *E. coli* BL21 (DE3) that may be active toward 1-dodecanol (Fig. 4C).

**Fig. 5:**
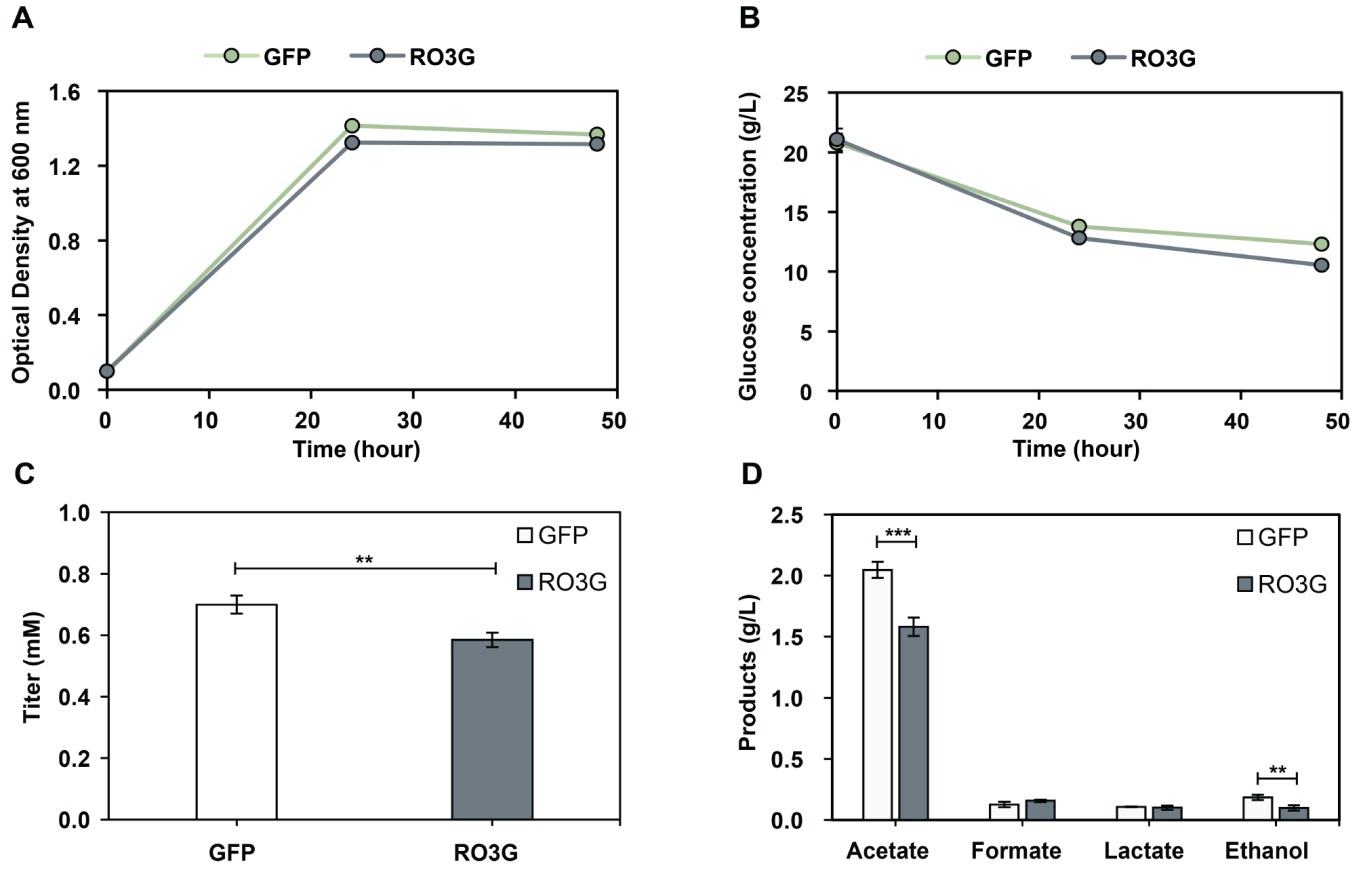
The expression of RO3G UDP-glycosyltransferase in *E. coli* BL21 (DE3) (**A**) Growth (**B**) Glucose consumption (**C**) 1-Dodecanol titer in the fed cultures (**D**) Fermentation products were observed from *E. coli* BL21 (DE3) harboring pCDF-GFP or pCDF-RO3G. Both strains were cultivated in M9 minimal medium with 2% (w/v) glucose fed with 2.0 mM 1-dodecanol. 1-Dodecanol and fermentation products were observed at 48 h after the inoculation. Asterisk (*) indicates *p*-values: * *p* ≤ 0.05, ** *p* ≤ 0.01 and *** *p* ≤ 0.005

The growth of *E. coli* BL21(DE3) expressing GFP and RO3G was not significantly different suggesting that the expression of RO3G did not impact cell growth and function (Fig. 5A). This aligned with the glucose consumption as the rates of glucose consumption were similar when compared between the two strains (Fig. 5B). Interestingly, in this feeding experiment with 2.0 mM 1-dodecanol, only 0.58 ± 0.02–0.70 ± 0.03 mM of 1-dodecanol was observed after 48 h of incubation (Fig. 5C). Although the volatility of 1-dodecanol, with a boiling point of 259 °C and low vapor pressure (0.0008 mmHg at 25 °C) according to PubChem (https://pubchem.ncbi.nlm.nih.gov/compound/Lauryl-Alcohol), suggests that it is not highly volatile under standard conditions. In this study, 1-dodecanol was suspended in water at a diluted concentration, which may reduce intermolecular forces and increase its volatility. Thus, a major portion of 1-dodecanol could be volatilized away from the cultures. This consideration is critical for optimizing downstream processes for the production of both 1-dodecanol and lauryl glucoside. Fermentation products (acetate, formate, ethanol and lactate) were also investigated after 48 h of incubation. The results suggest that acetate, which is the most dominant fermentation product, was fermented in a significantly lower amount (1.58 ± 0.08 g/L) compared to that of the GFP strain (2.05 ± 0.07 g/L). Similarly, ethanol was synthesized in significantly lower amounts in the GFP strain. These observations suggest that the expression of the heterologous UDP-glycosyltransferase may have redirected metabolic flux away from acetate and ethanol production. However, the amounts of formate and lactate did not differ significantly between the strains (Fig. 5D).

## Discussion

By designing novel biosynthetic pathways, it becomes feasible to produce non-native chemicals within microbial cells (Cho et al. 2022). Yet, one of the limitations that holds back the development of this concept is the lack of knowledge regarding the enzymes responsible for each biosynthetic step. Computational-guided methods have been employed to annotate hypothetical proteins, leveraging nature’s offerings by assigning functions to reported protein sequences. Several pipelines have been developed to predict the functions of various groups of proteins, with a particular focus on hypothetical proteins (Shahbaaz et al. 2013, 2015). Our previous work also developed a workflow to annotate enzymes and proteins from microalgal genomes for wastewater treatment as a library for synthetic biology (Uttarotai et al. 2022). Several other examples have been demonstrated including a very recent work that utilized a similar bioinformatic approach to predict the function of the hypothetical proteins from *Candida albicans* (Tripathi et al. 2024). In this study, we developed a computational workflow to predict enzyme functions, specifically targeting UDP-glycosyltransferases with activity toward 1-dodecanol.

Webservers to predict the function of the proteins are available, in this work, three well-reputed webservers; InterProScan, SUPERFAMILY 2.0 and CATH, were used. These webservers have previously been comparatively used in several studies (Shahbaaz et al. 2015; Uttarotai et al. 2022). InterProScan is known for the integrative classification, utilizing multiple databases for the comprehensive annotation (Blum et al. 2021), whereas SUPERFAMILY 2.0 focuses on structural classification based on the SCOP database, excelling in identifying evolutionary relationships and domain architecture, though it provides less detailed functional annotations (Pandurangan et al. 2019). CATH classifies protein domains hierarchically, emphasizing structure, architecture, topology, and homology, making it ideal for structural insights. CATH was demonstrated to produce relatively specific annotation (Uttarotai et al. 2022). Prediction of signal peptides is as well a crucial step for gene expression as the signal peptide induces the translocation of expressed proteins. The identification of the signal peptides in amino acid sequences has been made easy by a well-known web-based tool, SignalP (Teufel et al. 2022), and in the present work, UDP-glycosyltransferases should remain in the cell cytoplasm. Recently, protein structure modeling has advanced significantly, including the development of AI-based tools like Alphafold2. In this work, ColabFold (Mirdita et al. 2022), which was developed based on the well-known Alphafold, was used as it was more user-friendly and easily accessible. The ability to predict protein 3D structures from amino acid sequences enables further characterization of proteins, including molecular docking, which allows for the prediction of protein-protein or protein-ligand interactions (Chen et al. 2020). These tools particularly benefit the discovery of enzymes as reported previously (Papanikolaou et al. 2023; Zou et al. 2023).

UDP-glycosyltransferases are a major group of enzymes present in nature (Meech et al. 2019). This group of enzymes catalyzes the transfer of a sugar moiety from UDP-sugar donor to various functional groups (most frequently hydroxyl, carboxyl, or amine). Their native roles involve in the post-translational modification of eukaryotic organisms and the modification of xenobiotics (M. Wang et al. 2023a). This group of enzymes has been used in several novel synthetic pathways (Sattayawat et al. 2020; Wang et al. 2020; He et al. 2022); however, UDP-glycosyltransferases that are able to catalyze 1-dodecanol have never been reported. Genomes of four genera — *Candida*, *Pichia*, *Rhizopus*, and *Thermotoga* — were selected for genome mining due to their reported synthesis of lauryl glucoside. Function prediction using webservers was performed on all 240 UDP-glycosyltransferase candidates retrieved from the genomes of these organisms to narrow down the candidates. To facilitate downstream processes, subcellular localization was predicted to select candidates that tend to remain in the cytoplasm. Given the low water solubility of 1-dodecanol, a portion may bind to the phospholipid bilayer of the membrane. This could suggest that membrane-associated UDP-glycosyltransferases might be more effective. However, 1-dodecanol is also anticipated to be synthesized in the cytoplasm, where it may exist in free or micellar forms. Therefore, focusing on cytoplasmic UDP-glycosyltransferases may offer a more practical solution for the immediate conversion of 1-dodecanol. This approach helps avoid the formation of undesirable inclusion bodies, which can occur when expressing cell membrane-binding proteins (Bhatwa et al. 2021). Consequently, using soluble enzymes will optimize lauryl glucoside production in *E. coli*. Amino acid sequence alignment, 3D structure prediction and molecular docking were performed and one candidate, namely RO3G (Accession no. EIE92684.1), was selected. RO3G was further expressed in *E. coli* BL21 (DE3) and characterized for its ability to synthesize lauryl glucoside from 1-dodecanol. As a result, lauryl glucoside was synthesized through a feeding experiment confirming the activity of RO3G. Interestingly, LC-MS results revealed a small peak of lauryl glucoside in the *E. coli* strain expressing only GFP. Although the quantification indicated only a minimal amount of lauryl glucoside, this observation suggests that *E. coli* BL21(DE3) may possess some glycosyltransferase activity. *E. coli* is known to have glycosyltransferases involved in important cellular processes, such as the biosynthesis of O-antigen in lipopolysaccharides (LPS) (Brockhausen et al. 2008), peptidoglycan assembly (Mohammadi et al. 2007), and capsular polysaccharide formation (Lidholt et al. 1994). To determine whether these native glycosyltransferases might be similar to RO3G, we conducted an amino acid sequence alignment of all glycosyltransferases reported in *E. coli* BL21(DE3) and RO3G. Our analysis indicated that the native glycosyltransferases in *E. coli* do not possess the UDP-glycosyltransferase (UGT) conserved motif (Supplementary Data S2), suggesting that they are not UDP-glycosyltransferases. This implies that the observed formation of lauryl glucoside in *E. coli* may be due to other types of glycosyltransferases that utilize substrates other than UDP-glucose. Moreover, it was shown that the expression of this enzyme did not impose a burden on the cells, indicating that it was non-toxic and suitable for further use. Here, we also provide the fermentation profiles of the UDP-glycosyltransferase-expressing strain compared to the control, which, from a metabolic engineering perspective, understanding the formation of *E. coli* fermentation products is crucial for optimizing engineering strategies. Notably, it was significant that the production of acetate and ethanol was reduced, indicating a redirection of metabolic flux from these products toward the synthesis of lauryl glucoside since UDP-glycosyltransferase for the production of lauryl glucoside requires native UDP-glucose as the other substrate apart from 1-dodecanol. The utilization of available UDP-glucose redirects the carbon flux from acetyl-CoA as a common precursor of acetate and ethanol (Vivijs et al. 2015). This observation underscores the efficiency of UDP-glycosyltransferase in channeling metabolic resources into desired products. However, it should be taken into consideration that to validate the computational workflow, more than one selected candidate may be required in the experiments but in this work, as the purpose was to demonstrate that the computational predictions could identify a novel UDP-glycosyltransferase with promising activity, so only one candidate was further selected.

Discovering a UDP-glycosyltransferase active toward 1-dodecanol marks a significant step, however the enzyme’s specificity remains a critical factor for effective production. In this work, the low amount of lauryl glucoside synthesized suggests that RO3G has low specificity toward 1-dodecanol. UDP-glycosyltransferases constitute a large group of enzymes with a wide range of substrate specificities, and protein engineering offers a promising avenue to enhance their specificity toward desired substrates. Plant UDP-glycosyltransferases have previously been engineered to increase specificity (Wang et al. 2023a). For example, a recent work successfully engineered a UDP-glycosyltransferase to increase the specificity of UDP sugar donor to UDP-xylose (Wang et al. 2023b). In the future, detailed kinetic analyses of the UDP-glycosyltransferase will be essential to fully validate the workflow developed in this study and provide in-depth insights into the enzyme’s substrate specificity and catalytic efficiency. In this work, the newly discovered UDP-glycosyltransferase demonstrated activity toward 1-dodecanol, indicating its potential. Protein engineering can further increase this specificity, ultimately resulting in higher production yields. Altogether, the workflow presented here enables the discovery of UDP-glycosyltransferases active toward 1-dodecanol, with lauryl glucoside production successfully observed in the engineered *E. coli*.

## Conclusion

In this work, computational-guided discovery of C12 alcohol-active UDP-glycosyltransferases was implemented for lauryl glucoside production. Genomes of *Candida*, *Pichia*, *Rhizopus*, and *Thermotoga* were mined for UDP-glycosyltransferase genes as they were reported to synthesize lauryl glucoside. A total of 240 amino acid sequences were obtained and narrowed down to 176 using web-based function prediction tools, this number was further reduced to only 105 sequences without signal peptides. After the sequence alignment investigating the conserved catalytic function domains revealed 8 sequences with confirmed catalytic regions by which all of them were from *Rhizopus* genus. These sequences were used to model for their 3D structures and were subjected to molecular docking. One potential candidate was selected and expressed in *E. coli*. A feeding experiment with 1-dodecanol was performed and confirmed the production of lauryl glucoside. Altogether, a UDP-glycosyltransferase with activity toward 1-dodecanol was discovered and reported here.

## Electronic supplementary material

Below is the link to the electronic supplementary material.

Supplementary Material 1

Supplementary Material 2

## Data Availability

All data that support the findings of this study are included within this paper/supplementary files.
