# Supplementary material for: Computational-guided discovery of UDP-glycosyltransferases for lauryl glucoside production using engineered E. coli
Source: Bioresour Bioprocess. 2024 Oct 26;11(1):103. doi: 10.1186/s40643-024-00820-1 (PMC11561197; doi:10.1186/s40643-024-00820-1)
Supplement: Supplementary file 1 — Supplementary Material 1 [file 40643_2024_820_MOESM1_ESM.docx]

**Supplementary information**

**Computational-guided Discovery of UDP-glycosyltransferases for Lauryl Glucoside Production using Engineered *E. coli***

**Table S1** List of bioinformatic webtools used in this study

| **Software** | **URL** | **Remark** |
| --- | --- | --- |
| BLAST: Basic Local Alignment Search Tool | https://www.ncbi.nlm.nih.gov/ | BLASTp is used to search for similar sequences from microalgal genomes |
| Glycosyltransferase database | https://www.arabidopsis.org/browse/genefamily/Glycosyltransferase.jsp | Arabidopsis Glycosyltransferase Family 1 database |
| PubChem databank | https://pubchem.ncbi.nlm.nih.gov | 1-Dodecanol structure |
| InterProScan | http://www.ebi.ac.uk/InterProScan/ | Function prediction |
| SUPERFAMILY 2.0 | http://supfam.org | Function prediction |
| CATH | https://www.cathdb.info/ | Function prediction |
| Clustal Omega | https://www.ebi.ac.uk/jdispatcher/msa/clustalo | Amino acid sequence alignment |
| Alphafold2 | https://colab.research.google.com/ github/sokrypton/ColabFold/blob/main/AlphaFold2.ipynb #scrollTo=33g5IIegij5R | Structure modelling |
| Procheck | https://saves.mbi.ucla.edu/ | Protein structure validation |
| ModelArchive | https://www.modelarchive.org/ | Protein structure deposition |

**Table S2** Function and signal peptide predictions using different webservers

| **Description** | **Accession no.** | **Interpro** | **SuperFam** | **CATH** | **SignalP** |
| --- | --- | --- | --- | --- | --- |
| hypothetical protein G6F68_012122 [Rhizopus microsporus] | KAG1251748.1 | UDP-glycosyltransferase activity (GO:0008194) | UDP-Glycosyltransferase/glycogen phosphorylase | UDP-glucuronosyltransferase (3.40.50.2000/FF/21) | 1.000025 |
| hypothetical protein G6F71_009437 [Rhizopus microsporus] | KAG1167669.1 | UDP-glycosyltransferase activity (GO:0008194) | UDP-Glycosyltransferase/glycogen phosphorylase | UDP-glucuronosyltransferase (3.40.50.2000/FF/21) | 1.000056 |
| hypothetical protein RMCBS344292_17089 [Rhizopus microsporus] | CEJ03099.1 | UDP-glycosyltransferase activity (GO:0008194) | UDP-Glycosyltransferase/glycogen phosphorylase | UDP-glucuronosyltransferase (3.40.50.2000/FF/21) | 0.000267 |
| hypothetical protein RMATCC62417_06715 [Rhizopus microsporus] | CEG70897.1 | UDP-glycosyltransferase activity (GO:0008194) | UDP-Glycosyltransferase/glycogen phosphorylase | UDP-glucuronosyltransferase (3.40.50.2000/FF/21) | 0.000261 |
| hypothetical protein RMCBS344292_04015 [Rhizopus microsporus] | CEI89665.1 | UDP-glycosyltransferase activity (GO:0008194) | UDP-Glycosyltransferase/glycogen phosphorylase | UDP-glucuronosyltransferase (3.40.50.2000/FF/21) | 0.000247 |
| hypothetical protein RMCBS344292_04012 [Rhizopus microsporus] | CEI89662.1 | UDP-glycosyltransferase activity (GO:0008194) | UDP-Glycosyltransferase/glycogen phosphorylase | UDP-glucuronosyltransferase (3.40.50.2000/FF/21) | 0.000248 |
| hypothetical protein RMCBS344292_04028 [Rhizopus microsporus] | CEI89679.1 | UDP-glycosyltransferase activity (GO:0008194) | UDP-Glycosyltransferase/glycogen phosphorylase | UDP-glucuronosyltransferase (3.40.50.2000/FF/21) | 0.000234 |
| hypothetical protein RMCBS344292_04015 [Rhizopus microsporus] | ORE06707.1 | UDP-glycosyltransferase activity (GO:0008194) | UDP-Glycosyltransferase/glycogen phosphorylase | UDP-glucuronosyltransferase (3.40.50.2000/FF/21) | 0.000226 |
| hypothetical protein RMCBS344292_04012 [Rhizopus microsporus] | KAG1394693.1 | UDP-glycosyltransferase activity (GO:0008194 | UDP-Glycosyltransferase/glycogen phosphorylase | UDP-glucuronosyltransferase (3.40.50.2000/FF/50) | 0.000412 |
| hypothetical protein CU098_000159 [Rhizopus stolonifer] | KAG0931570.1 | UDP-glycosyltransferase activity (GO:0008194 | UDP-Glycosyltransferase/glycogen phosphorylase | UDP-glucuronosyltransferase (3.40.50.2000/FF/50) | 1.000075 |
| hypothetical protein G6F56_001855 [Rhizopus delemar] | KAG0850478.1 | UDP-glycosyltransferase activity (GO:0008194 | UDP-Glycosyltransferase/glycogen phosphorylase | UDP-glucuronosyltransferase (3.40.50.2000/FF/21) | 1.000075 |
| hypothetical protein RMATCC62417_06715 [Rhizopus microsporus] | KAG1050499.1 | UDP-glycosyltransferase activity (GO:0008194) | UDP-Glycosyltransferase/glycogen phosphorylase | UDP-glucuronosyltransferase (3.40.50.2000/FF/21) | 0.027979 |
| hypothetical protein RMCBS344292_17089 [Rhizopus microsporus] | KAG1272136.1 | UDP-glycosyltransferase activity (GO:0008194) | UDP-Glycosyltransferase/glycogen phosphorylase | UDP-glucuronosyltransferase (3.40.50.2000/FF/50) | 1.000075 |
| hypothetical protein RMCBS344292_04028 [Rhizopus microsporus] | KAG1302451.1 | UDP-glycosyltransferase activity (GO:0008194) | UDP-Glycosyltransferase/glycogen phosphorylase | UDP-glucuronosyltransferase (3.40.50.2000/FF/21) | 1.000029 |
| hypothetical protein RMATCC62417_06715 [Rhizopus microsporus] | KAG1139238.1 | UDP-glycosyltransferase activity (GO:0008194) | UDP-Glycosyltransferase/glycogen phosphorylase | UDP-glucuronosyltransferase (3.40.50.2000/FF/21) | 0.000236 |
| hypothetical protein RMCBS344292_17089 [Rhizopus microsporus] | KAG1411923.1 | UDP-glycosyltransferase activity (GO:0008194) | UDP-Glycosyltransferase/glycogen phosphorylase | UDP-glucuronosyltransferase (3.40.50.2000/FF/50) | 0.000412 |
| hypothetical protein RMCBS344292_04015 [Rhizopus microsporus] | KAG1373292.1 | UDP-glycosyltransferase activity (GO:0008194) | UDP-Glycosyltransferase/glycogen phosphorylase | UDP-glucuronosyltransferase (3.40.50.2000/FF/50) | 0.000412 |
| hypothetical protein G6F60_010696 [Rhizopus arrhizus] | KAG1044389.1 | UDP-glycosyltransferase activity (GO:0008194) | UDP-Glycosyltransferase/glycogen phosphorylase | UDP-glucuronosyltransferase (3.40.50.2000/FF/21) | 0.000291 |
| hypothetical protein G6F62_011710 [Rhizopus arrhizus] | KAG0804981.1 | UDP-glycosyltransferase activity (GO:0008194) | UDP-Glycosyltransferase/glycogen phosphorylase | UDP-glucuronosyltransferase (3.40.50.2000/FF/21) | 0.005701 |
| hypothetical protein G6F66_010418 [Rhizopus arrhizus] | KAG1623161.1 | UDP-glycosyltransferase activity (GO:0008194) | UDP-Glycosyltransferase/glycogen phosphorylase | UDP-glucuronosyltransferase (3.40.50.2000/FF/21) | 0.395265 |
| hypothetical protein G6F31_016749 [Rhizopus arrhizus] | KAG1493412.1 | UDP-glycosyltransferase activity (GO:0008194) | UDP-Glycosyltransferase/glycogen phosphorylase | UDP-glucuronosyltransferase (3.40.50.2000/FF/21) | 1.00005 |
| hypothetical protein G6F17_009845 [Rhizopus arrhizus] | KAG1107512.1 | UDP-glycosyltransferase activity (GO:0008194) | UDP-Glycosyltransferase/glycogen phosphorylase | UDP-glucuronosyltransferase (3.40.50.2000/FF/50) | 0.069754 |
| hypothetical protein G6F43_007235 [Rhizopus delemar] | KAG1494360.1 | UDP-glycosyltransferase activity (GO:0008194) | UDP-Glycosyltransferase/glycogen phosphorylase | UDP-glucuronosyltransferase (3.40.50.2000/FF/21) | 0.000267 |
| hypothetical protein G6F66_013406 [Rhizopus arrhizus] | EIE92684.1 | UDP-glycosyltransferase activity (GO:0008194) | UDP-Glycosyltransferase/glycogen phosphorylase | UDP-glucuronosyltransferase (3.40.50.2000/FF/21) | 1.000065 |
| hypothetical protein RO3G_14936 [Rhizopus delemar RA 99-880] | EIE90241.1 | UDP-glycosyltransferase activity (GO:0008194) | UDP-Glycosyltransferase/glycogen phosphorylase | UDP-glucuronosyltransferase (3.40.50.2000/FF/21) | 0.000267 |
| hypothetical protein G6F64_010917 [Rhizopus arrhizus] | KAG1646302.1 | UDP-glycosyltransferase activity (GO:0008194) | UDP-Glycosyltransferase/glycogen phosphorylase | UDP-glucuronosyltransferase (3.40.50.2000/FF/21) | 0.022733 |
| hypothetical protein G6F38_009979 [Rhizopus arrhizus] | KAG1034485.1 | UDP-glycosyltransferase activity (GO:0008194) | UDP-Glycosyltransferase/glycogen phosphorylase | UDP-glucuronosyltransferase (3.40.50.2000/FF/21) | 1.000057 |
| hypothetical protein G6F48_005792 [Rhizopus delemar] | EIE90240.1 | UDP-glycosyltransferase activity (GO:0008194) | UDP-Glycosyltransferase/glycogen phosphorylase | UDP-glucuronosyltransferase (3.40.50.2000/FF/21) | 1.000065 |
| hypothetical protein G6F58_008295 [Rhizopus delemar] | KAG1257709.1 | UDP-glycosyltransferase activity (GO:0008194) | UDP-Glycosyltransferase/glycogen phosphorylase | UDP-glucuronosyltransferase (3.40.50.2000/FF/21) | 0.002017 |
| hypothetical protein G6F61_010306 [Rhizopus arrhizus] | KAG1140655.1 | UDP-glycosyltransferase activity (GO:0008194) | UDP-Glycosyltransferase/glycogen phosphorylase | UDP-glucuronosyltransferase (3.40.50.2000/FF/21) | 0.000252 |
| hypothetical protein G6F21_005373 [Rhizopus arrhizus] | KAG0927131.1 | UDP-glycosyltransferase activity (GO:0008194) | UDP-Glycosyltransferase/glycogen phosphorylase | UDP-glucuronosyltransferase (3.40.50.2000/FF/50) | 0.000942 |
| hypothetical protein G6F33_012713 [Rhizopus arrhizus] | KAG1506235.1 | UDP-glycosyltransferase activity (GO:0008194) | UDP-Glycosyltransferase/glycogen phosphorylase | UDP-glucuronosyltransferase (3.40.50.2000/FF/21) | 0.00439 |
| hypothetical protein G6F43_011462 [Rhizopus delemar] | KAG1540849.1 | UDP-glycosyltransferase activity (GO:0008194) | UDP-Glycosyltransferase/glycogen phosphorylase | UDP-glucuronosyltransferase (3.40.50.2000/FF/21) | 1.000065 |
| hypothetical protein G6F20_012271 [Rhizopus arrhizus] | KAG1448890.1 | UDP-glycosyltransferase activity (GO:0008194) | UDP-Glycosyltransferase/glycogen phosphorylase | UDP-glucuronosyltransferase (3.40.50.2000/FF/50) | 0.000662 |
| hypothetical protein G6F45_010994 [Rhizopus arrhizus] | KAG1487268.1 | UDP-glycosyltransferase activity (GO:0008194) | UDP-Glycosyltransferase/glycogen phosphorylase | UDP-glucuronosyltransferase (3.40.50.2000/FF/50) | 1.00005 |
| hypothetical protein G6F53_012760 [Rhizopus delemar] | KAG0847242.1 | UDP-glycosyltransferase activity (GO:0008194) | UDP-Glycosyltransferase/glycogen phosphorylase | UDP-glucuronosyltransferase (3.40.50.2000/FF/50) | 0.000942 |
| hypothetical protein G6F40_009922 [Rhizopus arrhizus] | KAG1033902.1 | UDP-glycosyltransferase activity (GO:0008194) | UDP-Glycosyltransferase/glycogen phosphorylase | UDP-glucuronosyltransferase (3.40.50.2000/FF/50) | 0.063059 |
| hypothetical protein G6F53_012591 [Rhizopus delemar] | KAG1532670.1 | UDP-glycosyltransferase activity (GO:0008194) | UDP-Glycosyltransferase/glycogen phosphorylase | UDP-glucuronosyltransferase (3.40.50.2000/FF/50) | 1.000031 |
| hypothetical protein RO3G_17395 [Rhizopus delemar RA 99-880] | KAG1300452.1 | UDP-glycosyltransferase activity (GO:0008194) | UDP-Glycosyltransferase/glycogen phosphorylase | UDP-glucuronosyltransferase (3.40.50.2000/FF/50) | 0.000942 |
| hypothetical protein G6F64_005283 [Rhizopus arrhizus] | EIE90221.1 | UDP-glycosyltransferase activity (GO:0008194) | UDP-Glycosyltransferase/glycogen phosphorylase | UDP-glucuronosyltransferase (3.40.50.2000/FF/21) | 0.000409 |
| hypothetical protein RO3G_14952 [Rhizopus delemar RA 99-880] | KAG1489076.1 | UDP-glycosyltransferase activity (GO:0008194) | UDP-Glycosyltransferase/glycogen phosphorylase | UDP-glucuronosyltransferase (3.40.50.2000/FF/21) | 0.000409 |
| hypothetical protein G6F44_000947 [Rhizopus delemar] | KAG1341303.1 | UDP-glycosyltransferase activity (GO:0008194) | UDP-Glycosyltransferase/glycogen phosphorylase | UDP-glucuronosyltransferase (3.40.50.2000/FF/50) | 0.000277 |
| hypothetical protein G6F43_013399 [Rhizopus delemar] | KAG1319811.1 | UDP-glycosyltransferase activity (GO:0008194) | UDP-Glycosyltransferase/glycogen phosphorylase | UDP-glucuronosyltransferase (3.40.50.2000/FF/21) | 0.000942 |
| hypothetical protein RO3G_14951 [Rhizopus delemar RA 99-880] | KAG1614468.1 | UDP-glycosyltransferase activity (GO:0008194) | UDP-Glycosyltransferase/glycogen phosphorylase | UDP-glucuronosyltransferase (3.40.50.2000/FF/50) | 0.00439 |
| hypothetical protein G6F68_009172 [Rhizopus microsporus] | KAG1443864.1 | UDP-glycosyltransferase activity (GO:0008194) | UDP-Glycosyltransferase/glycogen phosphorylase | UDP-glucuronosyltransferase (3.40.50.2000/FF/50) | 0.000267 |
| hypothetical protein G6F38_008956 [Rhizopus arrhizus] | KAG1487553.1 | UDP-glycosyltransferase activity (GO:0008194) | UDP-Glycosyltransferase/glycogen phosphorylase | UDP-glucuronosyltransferase (3.40.50.2000/FF/50) | 0.000267 |
| hypothetical protein RMCBS344292_04015 [Rhizopus microsporus] | KAG1509099.1 | UDP-glycosyltransferase activity (GO:0008194) | UDP-Glycosyltransferase/glycogen phosphorylase | UDP-glucuronosyltransferase (3.40.50.2000/FF/21) | 0.000221 |
| hypothetical protein G6F30_012831 [Rhizopus arrhizus] | KAG1541320.1 | UDP-glycosyltransferase activity (GO:0008194) | UDP-Glycosyltransferase/glycogen phosphorylase | UDP-glucuronosyltransferase (3.40.50.2000/FF/21) | 0.000221 |
| hypothetical protein G6F52_011945 [Rhizopus delemar] | KAG1445822.1 | UDP-glycosyltransferase activity (GO:0008194) | UDP-Glycosyltransferase/glycogen phosphorylase | UDP-glucuronosyltransferase (3.40.50.2000/FF/21) | 0.000221 |
| hypothetical protein G6F49_012027 [Rhizopus delemar] | KAG1497540.1 | UDP-glycosyltransferase activity (GO:0008194) | UDP-Glycosyltransferase/glycogen phosphorylase | UDP-glucuronosyltransferase (3.40.50.2000/FF/21) | 0.000221 |
| hypothetical protein G6F56_008814 [Rhizopus delemar] | AOC55050.1 | UDP-glycosyltransferase activity (GO:0008194) | UDP-Glycosyltransferase/glycogen phosphorylase | UDP-glucuronosyltransferase (3.40.50.2000/FF/21) | 0.000269 |
| hypothetical protein G6F54_012762 [Rhizopus delemar] | KAG0751170.1 | UDP-glycosyltransferase activity (GO:0008194) | UDP-Glycosyltransferase/glycogen phosphorylase | UDP-glucuronosyltransferase (3.40.50.2000/FF/50) | 1.000061 |
| hypothetical protein G6F17_012705 [Rhizopus arrhizus] | KAG1226554.1 | UDP-glycosyltransferase activity (GO:0008194) | UDP-Glycosyltransferase/glycogen phosphorylase | UDP-glucuronosyltransferase (3.40.50.2000/FF/50) | 0.000353 |
| hypothetical protein G6F25_010102 [Rhizopus arrhizus] | KAG1319493.1 | UDP-glycosyltransferase activity (GO:0008194) | UDP-Glycosyltransferase/glycogen phosphorylase | UDP-glucuronosyltransferase (3.40.50.2000/FF/50) | 1.000045 |
| hypothetical protein G6F51_012997 [Rhizopus arrhizus] | EIE79820.1 | UDP-glycosyltransferase activity (GO:0008194) | UDP-Glycosyltransferase/glycogen phosphorylase | UDP-glucuronosyltransferase (3.40.50.2000/FF/50) | 1.000038 |
| hypothetical protein G6F64_012688 [Rhizopus arrhizus] | EIE79839.1 | UDP-glycosyltransferase activity (GO:0008194) | UDP-Glycosyltransferase/glycogen phosphorylase | UDP-glucuronosyltransferase (3.40.50.2000/FF/50) | 1.000069 |
| hypothetical protein RO3G_14932 [Rhizopus delemar RA 99-880] | KAG0777203.1 | UDP-glycosyltransferase activity (GO:0008194) | UDP-Glycosyltransferase/glycogen phosphorylase | UDP-glucuronosyltransferase (3.40.50.2000/FF/50) | 1.000061 |
| hypothetical protein G6F54_011697 [Rhizopus delemar] | KAG1137843.1 | UDP-glycosyltransferase activity (GO:0008194) | UDP-Glycosyltransferase/glycogen phosphorylase | UDP-glucuronosyltransferase (3.40.50.2000/FF/50) | 1.000085 |
| hypothetical protein RMATCC62417_06715 [Rhizopus microsporus] | KAG1131436.1 | UDP-glycosyltransferase activity (GO:0008194) | UDP-Glycosyltransferase/glycogen phosphorylase | UDP-glucuronosyltransferase (3.40.50.2000/FF/50) | 1.000063 |
| hypothetical protein G6F62_005132 [Rhizopus arrhizus] | RCH96098.1 | UDP-glycosyltransferase activity (GO:0008194) | UDP-Glycosyltransferase/glycogen phosphorylase | UDP-glucuronosyltransferase (3.40.50.2000/FF/118) | 0.017798 |
| hypothetical protein G6F62_011661 [Rhizopus arrhizus] | KAG1450945.1 | UDP-glycosyltransferase activity (GO:0008194) | UDP-Glycosyltransferase/glycogen phosphorylase | UDP-glucuronosyltransferase (3.40.50.2000/FF/118) | 0.017798 |
| hypothetical protein G6F45_012617 [Rhizopus arrhizus] | RCH94079.1 | UDP-glycosyltransferase activity (GO:0008194) | UDP-Glycosyltransferase/glycogen phosphorylase | UDP-glucuronosyltransferase (3.40.50.2000/FF/21) | 0.000249 |
| hypothetical protein G6F55_012527 [Rhizopus delemar] | CEI99983.1 | UDP-glycosyltransferase activity (GO:0008194) | UDP-Glycosyltransferase/glycogen phosphorylase | UDP-glucuronosyltransferase (3.40.50.2000/FF/21) | 0.000574 |
| hypothetical protein G6F54_012586 [Rhizopus delemar] | CEG68700.1 | UDP-glycosyltransferase activity (GO:0008194) | UDP-Glycosyltransferase/glycogen phosphorylase | UDP-glucuronosyltransferase (3.40.50.2000/FF/21) | 0.000449 |
| hypothetical protein G6F52_011224 [Rhizopus delemar] | CEI93939.1 | UDP-glycosyltransferase activity (GO:0008194) | UDP-Glycosyltransferase/glycogen phosphorylase | UDP-glucuronosyltransferase (3.40.50.2000/FF/21) | 0.000269 |
| hypothetical protein G6F49_011915 [Rhizopus delemar] | KAG1174810.1 | UDP-glycosyltransferase activity (GO:0008194) | UDP-Glycosyltransferase/glycogen phosphorylase | UDP-glucuronosyltransferase (3.40.50.2000/FF/21) | 0.001883 |
| hypothetical protein G6F55_011800 [Rhizopus delemar] | CEG71252.1 | UDP-glycosyltransferase activity (GO:0008194) | UDP-Glycosyltransferase/glycogen phosphorylase | UDP-glucuronosyltransferase (3.40.50.2000/FF/21) | 1.000048 |
| hypothetical protein G6F53_011950 [Rhizopus delemar] | RCH95492.1 | UDP-glycosyltransferase activity (GO:0008194) | UDP-Glycosyltransferase/glycogen phosphorylase | UDP-glucuronosyltransferase (3.40.50.2000/FF/21) | 0.000263 |
| hypothetical protein RO3G_14946 [Rhizopus delemar RA 99-880] | CEI93940.1 | UDP-glycosyltransferase activity (GO:0008194) | UDP-Glycosyltransferase/glycogen phosphorylase | UDP-glucuronosyltransferase (3.40.50.2000/FF/21) | 0.000257 |
| hypothetical protein G6F66_012077 [Rhizopus arrhizus] | CEI88156.1 | UDP-glycosyltransferase activity (GO:0008194) | UDP-Glycosyltransferase/glycogen phosphorylase | UDP-glucuronosyltransferase (3.40.50.2000/FF/21) | 0.000313 |
| hypothetical protein G6F24_014574 [Rhizopus arrhizus] | KAG1171912.1 | UDP-glycosyltransferase activity (GO:0008194) | UDP-Glycosyltransferase/glycogen phosphorylase | UDP-glucuronosyltransferase (3.40.50.2000/FF/21) | 0.000313 |
| hypothetical protein G6F35_002874 [Rhizopus arrhizus] | RCI01321.1 | UDP-glycosyltransferase activity (GO:0008194) | UDP-Glycosyltransferase/glycogen phosphorylase | UDP-glucuronosyltransferase (3.40.50.2000/FF/21) | 0.000294 |
| hypothetical protein G6F62_011812 [Rhizopus arrhizus] | KAG1135664.1 | UDP-glycosyltransferase activity (GO:0008194) | UDP-Glycosyltransferase/glycogen phosphorylase | UDP-glucuronosyltransferase (3.40.50.2000/FF/21) | 1.000095 |
| hypothetical protein RO3G_04525 [Rhizopus delemar RA 99-880] | RCH95491.1 | UDP-glycosyltransferase activity (GO:0008194) | UDP-Glycosyltransferase/glycogen phosphorylase | UDP-glucuronosyltransferase (3.40.50.2000/FF/21) | 0.000267 |
| hypothetical protein G6F56_005627 [Rhizopus delemar] | KAG1278110.1 | UDP-glycosyltransferase activity (GO:0008194) | UDP-Glycosyltransferase/glycogen phosphorylase | UDP-glucuronosyltransferase (3.40.50.2000/FF/50) | 0.000437 |
| hypothetical protein RO3G_04544 [Rhizopus delemar RA 99-880] | CEI99486.1 | UDP-glycosyltransferase activity (GO:0008194) | UDP-Glycosyltransferase/glycogen phosphorylase | UDP-glucuronosyltransferase (3.40.50.2000/FF/21) | 0.000239 |
| hypothetical protein G6F21_013418 [Rhizopus arrhizus] | KAG1107114.1 | UDP-glycosyltransferase activity (GO:0008194) | UDP-Glycosyltransferase/glycogen phosphorylase | UDP-glucuronosyltransferase (3.40.50.2000/FF/118) | 0.000499 |
| hypothetical protein G6F56_008814 [Rhizopus delemar] | KAG1038511.1 | UDP-glycosyltransferase activity (GO:0008194) | UDP-Glycosyltransferase/glycogen phosphorylase | UDP-glucuronosyltransferase (3.40.50.2000/FF/21) | 1.000061 |
| hypothetical protein G6F37_013451 [Rhizopus arrhizus] | KAG0813888.1 | UDP-glycosyltransferase activity (GO:0008194) | UDP-Glycosyltransferase/glycogen phosphorylase | UDP-glucuronosyltransferase (3.40.50.2000/FF/21) | 0.000277 |
| hypothetical protein G6F38_013377 [Rhizopus arrhizus] | KAG0787672.1 | UDP-glycosyltransferase activity (GO:0008194) | UDP-Glycosyltransferase/glycogen phosphorylase | UDP-glucuronosyltransferase (3.40.50.2000/FF/21) | 0.000277 |
| hypothetical protein CU098_009727 [Rhizopus stolonifer] | KAG0962502.1 | UDP-glycosyltransferase activity (GO:0008194) | UDP-Glycosyltransferase/glycogen phosphorylase | UDP-glucuronosyltransferase (3.40.50.2000/FF/21) | 0.000277 |
| hypothetical protein G6F56_008214 [Rhizopus delemar] | KAG0825681.1 | UDP-glycosyltransferase activity (GO:0008194) | UDP-Glycosyltransferase/glycogen phosphorylase | UDP-glucuronosyltransferase (3.40.50.2000/FF/21) | 0.000277 |
| hypothetical protein G6F33_012713 [Rhizopus arrhizus] | KAG0865802.1 | UDP-glycosyltransferase activity (GO:0008194) | UDP-Glycosyltransferase/glycogen phosphorylase | UDP-glucuronosyltransferase (3.40.50.2000/FF/21) | 0.000277 |
| hypothetical protein G6F20_012271 [Rhizopus arrhizus] | KAG1289780.1 | UDP-glycosyltransferase activity (GO:0008194) | UDP-Glycosyltransferase/glycogen phosphorylase | UDP-glucuronosyltransferase (3.40.50.2000/FF/21) | 0.000277 |
| hypothetical protein G6F38_009979 [Rhizopus arrhizus] | EIE90219.1 | UDP-glycosyltransferase activity (GO:0008194) | UDP-Glycosyltransferase/glycogen phosphorylase | UDP-glucuronosyltransferase (3.40.50.2000/FF/21) | 1.000069 |
| hypothetical protein G6F64_010917 [Rhizopus arrhizus] | KAG1180901.1 | UDP-glycosyltransferase activity (GO:0008194) | UDP-Glycosyltransferase/glycogen phosphorylase | UDP-glucuronosyltransferase (3.40.50.2000/FF/21) | 0.000277 |
| hypothetical protein G6F31_016749 [Rhizopus arrhizus] | KAG1140388.1 | UDP-glycosyltransferase activity (GO:0008194) | UDP-Glycosyltransferase/glycogen phosphorylase | UDP-glucuronosyltransferase (3.40.50.2000/FF/21) | 1.000065 |
| hypothetical protein CU097_002135 [Rhizopus azygosporus] | KAG1466351.1 | UDP-glycosyltransferase activity (GO:0008194) | UDP-Glycosyltransferase/glycogen phosphorylase | UDP-glucuronosyltransferase (3.40.50.2000/FF/21) | 0.000187 |
| hypothetical protein G6F17_009845 [Rhizopus arrhizus] | KAG1646307.1 | UDP-glycosyltransferase activity (GO:0008194) | UDP-Glycosyltransferase/glycogen phosphorylase | UDP-glucuronosyltransferase (3.40.50.2000/FF/21) | 0.000187 |
| hypothetical protein G6F25_010102 [Rhizopus arrhizus] | KAG1571406.1 | UDP-glycosyltransferase activity (GO:0008194) | UDP-Glycosyltransferase/glycogen phosphorylase | UDP-glucuronosyltransferase (3.40.50.2000/FF/21) | 1.000056 |
| hypothetical protein RMCBS344292_08163 [Rhizopus microsporus] | KAG0846790.1 | UDP-glycosyltransferase activity (GO:0008194) | UDP-Glycosyltransferase/glycogen phosphorylase | UDP-glucuronosyltransferase (3.40.50.2000/FF/50) | 1.000056 |
| hypothetical protein G6F66_013406 [Rhizopus arrhizus] | KAG1489094.1 | UDP-glycosyltransferase activity (GO:0008194) | UDP-Glycosyltransferase/glycogen phosphorylase | UDP-glucuronosyltransferase (3.40.50.2000/FF/50) | 1.000056 |
| hypothetical protein G6F43_007235 [Rhizopus delemar] | RCH78013.1 | UDP-glycosyltransferase activity (GO:0008194) | UDP-Glycosyltransferase/glycogen phosphorylase | UDP-glucuronosyltransferase (3.40.50.2000/FF/21) | 1.00005 |
| hypothetical protein G6F71_004617 [Rhizopus microsporus] | KAG1120478.1 | UDP-glycosyltransferase activity (GO:0008194) | UDP-Glycosyltransferase/glycogen phosphorylase | UDP-glucoronosyl and UDP-glucosyl transferase family protein, putative (3.40.50.2000/FF/693) | 1.000026 |
| hypothetical protein RMATCC62417_07011 [Rhizopus microsporus] | KAG1139956.1 | UDP-glycosyltransferase activity (GO:0008194) | UDP-Glycosyltransferase/glycogen phosphorylase | UDP-glucuronosyltransferase (3.40.50.2000/FF/21) | 1.000063 |
| hypothetical protein G6F21_005373 [Rhizopus arrhizus] | KAG1080465.1 | UDP-glycosyltransferase activity (GO:0008194) | UDP-Glycosyltransferase/glycogen phosphorylase | UDP-glucuronosyltransferase (3.40.50.2000/FF/21) | 0.999353 |
| hypothetical protein CU097_012410 [Rhizopus azygosporus] | RCI02354.1 | UDP-glycosyltransferase activity (GO:0008194) | UDP-Glycosyltransferase/glycogen phosphorylase | UDP-glucuronosyltransferase (3.40.50.2000/FF/21) | 0.000207 |
| hypothetical protein RMCBS344292_08164 [Rhizopus microsporus] | KAG1227489.1 | UDP-glycosyltransferase activity (GO:0008194) | UDP-Glycosyltransferase/glycogen phosphorylase | UDP-glucuronosyltransferase (3.40.50.2000/FF/21) | 0.99985 |
| hypothetical protein G6F62_011710 [Rhizopus arrhizus] | XP_023461080.1 | hexosyltransferase activity (GO:0016758) UDP-glycosyltransferase activity (GO:0008194) | UDP-Glycosyltransferase/glycogen phosphorylase | Sterol 3-beta-glucosyltransferase (3.40.50.2000/FF/29) | 1.000018 |
| hypothetical protein RMCBS344292_02555 [Rhizopus microsporus] | RCH93946.1 | hexosyltransferase activity (GO:0016758) UDP-glycosyltransferase activity (GO:0008194) | UDP-Glycosyltransferase/glycogen phosphorylase | Sterol 3-beta-glucosyltransferase (3.40.50.2000/FF/29) | 1.000081 |
| hypothetical protein G6F40_009922 [Rhizopus arrhizus] | CEG67174.1 | UDP-glycosyltransferase activity (GO:0008194) hexosyltransferase activity (GO:0016758) | UDP-Glycosyltransferase/glycogen phosphorylase | Sterol 3-beta-glucosyltransferase (3.40.50.2000/FF/29) | 1.000057 |
| hypothetical protein G6F43_013399 [Rhizopus delemar] | KAG1170099.1 | UDP-glycosyltransferase activity (GO:0008194) hexosyltransferase activity (GO:0016758) | UDP-Glycosyltransferase/glycogen phosphorylase | Sterol 3-beta-glucosyltransferase (3.40.50.2000/FF/29) | 0.999704 |
| hypothetical protein RO3G_14932 [Rhizopus delemar RA 99-880] | CEG68474.1 | hexosyltransferase activity (GO:0016758) UDP-glycosyltransferase activity (GO:0008194) | UDP-Glycosyltransferase/glycogen phosphorylase | Sterol 3-beta-glucosyltransferase (3.40.50.2000/FF/29) | 0.999954 |
| hypothetical protein G6F54_011697 [Rhizopus delemar] | XP_023467596.1 | hexosyltransferase activity (GO:0016758) UDP-glycosyltransferase activity (GO:0008194 | UDP-Glycosyltransferase/glycogen phosphorylase | Sterol 3-beta-glucosyltransferase (3.40.50.2000/FF/29) | 1.000063 |
| hypothetical protein G6F71_006629 [Rhizopus microsporus] | CEG83847.1 | UDP-glycosyltransferase activity (GO:0008194) hexosyltransferase activity (GO:0016758) | UDP-Glycosyltransferase/glycogen phosphorylase | Sterol 3-beta-glucosyltransferase (3.40.50.2000/FF/29) | 1.000041 |
| hypothetical protein CU097_014263 [Rhizopus azygosporus] | RCH84061.1 | hexosyltransferase activity (GO:0016758) UDP-glycosyltransferase activity (GO:0008194) | UDP-Glycosyltransferase/glycogen phosphorylase | Sterol 3-beta-glucosyltransferase (3.40.50.2000/FF/29) | 1.000041 |
| hypothetical protein RMCBS344292_04015 [Rhizopus microsporus] | RCH97772.1 | UDP-glycosyltransferase activity (GO:0008194) hexosyltransferase activity (GO:0016758) | UDP-Glycosyltransferase/glycogen phosphorylase | Sterol 3-beta-glucosyltransferase (3.40.50.2000/FF/29) | 1.000008 |
| hypothetical protein G6F60_010696 [Rhizopus arrhizus] | RCI06016.1 | UDP-glycosyltransferase activity (GO:0008194) hexosyltransferase activity (GO:0016758) | UDP-Glycosyltransferase/glycogen phosphorylase | Sterol 3-beta-glucosyltransferase (3.40.50.2000/FF/29) | 1.000046 |
| hypothetical protein G6F38_012623 [Rhizopus arrhizus] | KAG1056023.1 | UDP-glycosyltransferase activity (GO:0008194) hexosyltransferase activity (GO:0016758) | UDP-Glycosyltransferase/glycogen phosphorylase | Sterol 3-beta-glucosyltransferase (3.40.50.2000/FF/29) | 1.000052 |
| hypothetical protein G6F64_005283 [Rhizopus arrhizus] | KAG1463282.1 | UDP-glycosyltransferase activity (GO:0008194) hexosyltransferase activity (GO:0016758) | UDP-Glycosyltransferase/glycogen phosphorylase | Sterol 3-beta-glucosyltransferase (3.40.50.2000/FF/29) | 1.000052 |
| hypothetical protein G6F62_005132 [Rhizopus arrhizus] | CEI96139.1 | hexosyltransferase activity (GO:0016758) UDP-glycosyltransferase activity (GO:0008194) | UDP-Glycosyltransferase/glycogen phosphorylase | Sterol 3-beta-glucosyltransferase (3.40.50.2000/FF/29) | 1.000045 |
| hypothetical protein G6F66_010418 [Rhizopus arrhizus] | KAG1030797.1 | UDP-glycosyltransferase activity (GO:0008194) hexosyltransferase activity (GO:0016758) | UDP-Glycosyltransferase/glycogen phosphorylase | Sterol 3-beta-glucosyltransferase (3.40.50.2000/FF/29) | 1.000035 |
| hypothetical protein CU097_009456 [Rhizopus azygosporus] | KAG0810880.1 | UDP-glycosyltransferase activity (GO:0008194) hexosyltransferase activity (GO:0016758) | UDP-Glycosyltransferase/glycogen phosphorylase | Sterol 3-beta-glucosyltransferase (3.40.50.2000/FF/29) | 1.000035 |
| hypothetical protein G6F58_008295 [Rhizopus delemar] | KAG1139966.1 | UDP-glycosyltransferase activity (GO:0008194) hexosyltransferase activity (GO:0016758) | UDP-Glycosyltransferase/glycogen phosphorylase | Sterol 3-beta-glucosyltransferase (3.40.50.2000/FF/29) | 1.000035 |
| hypothetical protein G6F43_011462 [Rhizopus delemar] | KAG1170223.1 | UDP-glycosyltransferase activity (GO:0008194) hexosyltransferase activity (GO:0016758) | UDP-Glycosyltransferase/glycogen phosphorylase | Sterol 3-beta-glucosyltransferase (3.40.50.2000/FF/29) | 0.999942 |
| hypothetical protein G6F61_010306 [Rhizopus arrhizus] | KAG1156637.1 | UDP-glycosyltransferase activity (GO:0008194) hexosyltransferase activity (GO:0016758) | UDP-Glycosyltransferase/glycogen phosphorylase | Sterol 3-beta-glucosyltransferase (3.40.50.2000/FF/29) | 1.000035 |
| hypothetical protein G6F66_012138 [Rhizopus arrhizus] | KAG0762257.1 | hexosyltransferase activity (GO:0016758) UDP-glycosyltransferase activity (GO:0008194) | UDP-Glycosyltransferase/glycogen phosphorylase | Sterol 3-beta-glucosyltransferase (3.40.50.2000/FF/29) | 1.000035 |
| hypothetical protein RMCBS344292_13575 [Rhizopus microsporus] | KAG1472087.1 | hexosyltransferase activity (GO:0016758) UDP-glycosyltransferase activity (GO:0008194) | UDP-Glycosyltransferase/glycogen phosphorylase | Sterol 3-beta-glucosyltransferase (3.40.50.2000/FF/29) | 1.00006 |
| hypothetical protein G6F38_009979 [Rhizopus arrhizus] | KAG0943589.1 | UDP-glycosyltransferase activity (GO:0008194) hexosyltransferase activity (GO:0016758) | UDP-Glycosyltransferase/glycogen phosphorylase | Sterol 3-beta-glucosyltransferase (3.40.50.2000/FF/29) | 0.999775 |
| hypothetical protein G6F31_016749 [Rhizopus arrhizus] | KAG0765378.1 | UDP-glycosyltransferase activity (GO:0008194) hexosyltransferase activity (GO:0016758) | UDP-Glycosyltransferase/glycogen phosphorylase | Sterol 3-beta-glucosyltransferase (3.40.50.2000/FF/29) | 0.999775 |
| hypothetical protein G6F53_012760 [Rhizopus delemar] | KAG0742934.1 | UDP-glycosyltransferase activity (GO:0008194) hexosyltransferase activity (GO:0016758) | UDP-Glycosyltransferase/glycogen phosphorylase | Sterol 3-beta-glucosyltransferase (3.40.50.2000/FF/29) | 0.999775 |
| hypothetical protein G6F66_013406 [Rhizopus arrhizus] | KAG0786273.1 | UDP-glycosyltransferase activity (GO:0008194) hexosyltransferase activity (GO:0016758) | UDP-Glycosyltransferase/glycogen phosphorylase | Sterol 3-beta-glucosyltransferase (3.40.50.2000/FF/29) | 0.999961 |
| hypothetical protein RO3G_14951 [Rhizopus delemar RA 99-880] | KAG0782652.1 | UDP-glycosyltransferase activity (GO:0008194) hexosyltransferase activity (GO:0016758) | UDP-Glycosyltransferase/glycogen phosphorylase | Sterol 3-beta-glucosyltransferase (3.40.50.2000/FF/29) | 1.000035 |
| hypothetical protein G6F31_016749 [Rhizopus arrhizus] | KAG0939282.1 | UDP-glycosyltransferase activity (GO:0008194) hexosyltransferase activity (GO:0016758) | UDP-Glycosyltransferase/glycogen phosphorylase | Sterol 3-beta-glucosyltransferase (3.40.50.2000/FF/29) | 1.000035 |
| hypothetical protein G6F38_009979 [Rhizopus arrhizus] | KAG1373493.1 | UDP-glycosyltransferase activity (GO:0008194) hexosyltransferase activity (GO:0016758) | UDP-Glycosyltransferase/glycogen phosphorylase | Sterol 3-beta-glucosyltransferase (3.40.50.2000/FF/29) | 1.000035 |
| hypothetical protein G6F66_013406 [Rhizopus arrhizus] | KAG1006203.1 | UDP-glycosyltransferase activity (GO:0008194) hexosyltransferase activity (GO:0016758) | UDP-Glycosyltransferase/glycogen phosphorylase | Sterol 3-beta-glucosyltransferase (3.40.50.2000/FF/29) | 1.000035 |
| hypothetical protein G6F40_009922 [Rhizopus arrhizus] | KAG0979484.1 | UDP-glycosyltransferase activity (GO:0008194) hexosyltransferase activity (GO:0016758) | UDP-Glycosyltransferase/glycogen phosphorylase | Sterol 3-beta-glucosyltransferase (3.40.50.2000/FF/29) | 1.000035 |
| hypothetical protein G6F56_008814 [Rhizopus delemar] | KAG1474340.1 | UDP-glycosyltransferase activity (GO:0008194) hexosyltransferase activity (GO:0016758) | UDP-Glycosyltransferase/glycogen phosphorylase | Sterol 3-beta-glucosyltransferase (3.40.50.2000/FF/29) | 1.00002 |
| hypothetical protein G6F64_005283 [Rhizopus arrhizus] | KAG1049495.1 | UDP-glycosyltransferase activity (GO:0008194) hexosyltransferase activity (GO:0016758) | UDP-Glycosyltransferase/glycogen phosphorylase | Sterol 3-beta-glucosyltransferase (3.40.50.2000/FF/29) | 0.999835 |
| hypothetical protein G6F38_009979 [Rhizopus arrhizus] | KAG1455928.1 | UDP-glycosyltransferase activity (GO:0008194) hexosyltransferase activity (GO:0016758) | UDP-Glycosyltransferase/glycogen phosphorylase | Sterol 3-beta-glucosyltransferase (3.40.50.2000/FF/29) | 0.999835 |
| hypothetical protein G6F31_016749 [Rhizopus arrhizus] | KAG1638403.1 | UDP-glycosyltransferase activity (GO:0008194) hexosyltransferase activity (GO:0016758) | UDP-Glycosyltransferase/glycogen phosphorylase | Sterol 3-beta-glucosyltransferase (3.40.50.2000/FF/29) | 0.999835 |
| hypothetical protein G6F42_016485 [Rhizopus arrhizus] | KAG1146216.1 | hexosyltransferase activity (GO:0016758) UDP-glycosyltransferase activity (GO:0008194) | UDP-Glycosyltransferase/glycogen phosphorylase | Sterol 3-beta-glucosyltransferase (3.40.50.2000/FF/29) | 0.999727 |
| hypothetical protein G6F49_012027 [Rhizopus delemar] | ORE13115.1 | UDP-glycosyltransferase activity (GO:0008194) hexosyltransferase activity (GO:0016758) | UDP-Glycosyltransferase/glycogen phosphorylase | Sterol 3-beta-glucosyltransferase UGT80A2 (3.40.50.2000/FF/9) | 1.00005 |
| hypothetical protein G6F64_010917 [Rhizopus arrhizus] | EIE87900.1 | UDP-glycosyltransferase activity (GO:0008194) hexosyltransferase activity (GO:0016758) | UDP-Glycosyltransferase/glycogen phosphorylase | Sterol 3-beta-glucosyltransferase UGT80A2 (3.40.50.2000/FF/9) | 1.000063 |
| hypothetical protein RMATCC62417_06715 [Rhizopus microsporus] | RCI02105.1 | UDP-glycosyltransferase activity (GO:0008194) hexosyltransferase activity (GO:0016758) | UDP-Glycosyltransferase/glycogen phosphorylase | Sterol 3-beta-glucosyltransferase (3.40.50.2000/FF/29) | 1.00002 |
| hypothetical protein G6F53_012760 [Rhizopus delemar] | KAG1223788.1 | UDP-glycosyltransferase activity (GO:0008194) | UDP-Glycosyltransferase/glycogen phosphorylase | Sterol 3-beta-glucosyltransferase UGT80A2 (3.40.50.2000/FF/9) | 1.000048 |
| hypothetical protein RMCBS344292_04015 [Rhizopus microsporus] | ORE16489.1 | UDP-glycosyltransferase activity (GO:0008194) | UDP-Glycosyltransferase/glycogen phosphorylase | Sterol 3-beta-glucosyltransferase UGT80A2 (3.40.50.2000/FF/9) | 0.994162 |
| hypothetical protein RMCBS344292_04015 [Rhizopus microsporus] | KAG1062597.1 | UDP-glycosyltransferase activity (GO:0008194) | UDP-Glycosyltransferase/glycogen phosphorylase | Sterol 3-beta-glucosyltransferase UGT80A2 (3.40.50.2000/FF/9) | 1.000063 |
| hypothetical protein RMCBS344292_04012 [Rhizopus microsporus] | CEI96138.1 | UDP-glycosyltransferase activity (GO:0008194) | UDP-Glycosyltransferase/glycogen phosphorylase | Sterol 3-beta-glucosyltransferase UGT80A2 (3.40.50.2000/FF/9) | 1.00005 |
| hypothetical protein RMATCC62417_06715 [Rhizopus microsporus] | CEJ02292.1 | hexosyltransferase activity (GO:0016758) | UDP-Glycosyltransferase/glycogen phosphorylase | Sterol 3-beta-glucosyltransferase (2.30.29.30/FF/303) | 1.000081 |
| hypothetical protein RMCBS344292_17089 [Rhizopus microsporus] | KAG0804100.1 | UDP-glycosyltransferase activity (GO:0008194) | UDP-Glycosyltransferase/glycogen phosphorylase | UDP-glucuronosyltransferase (3.40.50.2000/FF/21) | 1.000071 |
| hypothetical protein G6F38_008956 [Rhizopus arrhizus] | KAG1202963.1 | UDP-glycosyltransferase activity (GO:0008194) | UDP-Glycosyltransferase/glycogen phosphorylase | UDP-glucuronosyltransferase (3.40.50.2000/FF/118) | 0.998476 |
| hypothetical protein G6F60_010696 [Rhizopus arrhizus] | KAG1343730.1 | UDP-glycosyltransferase activity (GO:0008194) | UDP-Glycosyltransferase/glycogen phosphorylase | UDP-glucuronosyltransferase (3.40.50.2000/FF/118) | 1.00007 |
| hypothetical protein G6F66_010418 [Rhizopus arrhizus] | KAG0763646.1 | UDP-glycosyltransferase activity (GO:0008194) | UDP-Glycosyltransferase/glycogen phosphorylase | UDP-glucuronosyltransferase (3.40.50.2000/FF/118) | 0.039415 |
| hypothetical protein G6F43_013399 [Rhizopus delemar] | KAG0739144.1 | UDP-glycosyltransferase activity (GO:0008194) | UDP-Glycosyltransferase/glycogen phosphorylase | UDP-glucuronosyltransferase (3.40.50.2000/FF/118) | 0.039415 |
| hypothetical protein G6F43_012678 [Rhizopus delemar] | EIE82227.1 | UDP-glycosyltransferase activity (GO:0008194) | UDP-Glycosyltransferase/glycogen phosphorylase | UDP-glucuronosyltransferase (3.40.50.2000/FF/118) | 1.000079 |
| hypothetical protein G6F43_007235 [Rhizopus delemar] | KAG1503203.1 | UDP-glycosyltransferase activity (GO:0008194) | UDP-Glycosyltransferase/glycogen phosphorylase | UDP-glucuronosyltransferase (3.40.50.2000/FF/21) | 0.00088 |
| hypothetical protein G6F61_010306 [Rhizopus arrhizus] | KAG1464069.1 | UDP-glycosyltransferase activity (GO:0008194) | UDP-Glycosyltransferase/glycogen phosphorylase | UDP-glucuronosyltransferase (3.40.50.2000/FF/21) | 0.00088 |
| hypothetical protein G6F62_011710 [Rhizopus arrhizus] | KAG1461069.1 | UDP-glycosyltransferase activity (GO:0008194) | UDP-Glycosyltransferase/glycogen phosphorylase | UDP-glucuronosyltransferase 2B14-like Protein (3.40.50.2000/FF/189) | 0.002248 |
| hypothetical protein G6F66_010418 [Rhizopus arrhizus] | KAG1164127.1 | UDP-glycosyltransferase activity (GO:0008194) | UDP-Glycosyltransferase/glycogen phosphorylase | UDP-glucuronosyltransferase (3.40.50.2000/FF/118) | 0.040421 |
| hypothetical protein RO3G_14951 [Rhizopus delemar RA 99-880] | KAG1151654.1 | UDP-glycosyltransferase activity (GO:0008194) | UDP-Glycosyltransferase/glycogen phosphorylase | UDP-glucuronosyltransferase (3.40.50.2000/FF/118) | 0.040421 |
| hypothetical protein G6F20_005215 [Rhizopus arrhizus] | RCI06813.1 | UDP-glycosyltransferase activity (GO:0008194) | UDP-Glycosyltransferase/glycogen phosphorylase | UDP-glucuronosyltransferase (3.40.50.2000/FF/21) | 1.000037 |
| hypothetical protein G6F22_007238 [Rhizopus arrhizus] | KAG1474332.1 | UDP-glycosyltransferase activity (GO:0008194) | UDP-Glycosyltransferase/glycogen phosphorylase | UDP-glucuronosyltransferase (3.40.50.2000/FF/21) | 0.000215 |
| hypothetical protein G6F31_008606 [Rhizopus arrhizus] | KAG1443981.1 | UDP-glycosyltransferase activity (GO:0008194) | UDP-Glycosyltransferase/glycogen phosphorylase | UDP-glucuronosyltransferase (3.40.50.2000/FF/50) | 1.000062 |
| hypothetical protein G6F19_009700 [Rhizopus arrhizus] | KAG1488691.1 | UDP-glycosyltransferase activity (GO:0008194) | UDP-Glycosyltransferase/glycogen phosphorylase | UDP-glucuronosyltransferase (3.40.50.2000/FF/21) | 0.000221 |
| hypothetical protein G6F16_009949 [Rhizopus arrhizus] | KAG1062151.1 | UDP-glycosyltransferase activity (GO:0008194) | UDP-Glycosyltransferase/glycogen phosphorylase | UDP-glucoronosyl and UDP-glucosyl transferase family protein, putative (3.40.50.2000/FF/693) | 1.000075 |
| uncharacterized protein C5L36_0A01280 [Pichia kudriavzevii] | XP_029318999.1 | UDP-glycosyltransferase activity (GO:0008194) hexosyltransferase activity (GO:0016758) | UDP-Glycosyltransferase/glycogen phosphorylase | Sterol 3-beta-glucosyltransferase (3.40.50.2000/FF/29) | 1.000055 |
| hypothetical protein CANINC_002767 [[Candida] inconspicua] | TID26235.1 | hexosyltransferase activity (GO:0016758) UDP-glycosyltransferase activity (GO:0008194) | UDP-Glycosyltransferase/glycogen phosphorylase | Sterol 3-beta-glucosyltransferase (3.40.50.2000/FF/29) | 1.000046 |
| hypothetical protein CAS74_001324 [Pichia kudriavzevii] | OUT23021.1 | UDP-glycosyltransferase activity (GO:0008194) hexosyltransferase activity (GO:0016758) | UDP-Glycosyltransferase/glycogen phosphorylase | Sterol 3-beta-glucosyltransferase (3.40.50.2000/FF/29) | 1.000045 |
| hypothetical protein PICMEDRAFT_34457 [Pichia membranifaciens... | XP_019016766.1 | UDP-glycosyltransferase activity (GO:0008194) hexosyltransferase activity (GO:0016758) | UDP-Glycosyltransferase/glycogen phosphorylase | Sterol 3-beta-glucosyltransferase (3.40.50.2000/FF/29) | 1.000038 |
| hypothetical protein PMKS-001236 [Pichia membranifaciens] | GAV27768.1 | UDP-glycosyltransferase activity (GO:0008194) hexosyltransferase activity (GO:0016758) | UDP-Glycosyltransferase/glycogen phosphorylase | Sterol 3-beta-glucosyltransferase (3.40.50.2000/FF/29) | 1.000041 |
| hypothetical protein JL09_g943 [Pichia kudriavzevii] | KGK39899.1 | hexosyltransferase activity (GO:0016758) UDP-glycosyltransferase activity (GO:0008194) | UDP-Glycosyltransferase/glycogen phosphorylase | Sterol 3-beta-glucosyltransferase (3.40.50.2000/FF/29) | 1.00007 |
| hypothetical protein JL09_g6539 [Pichia kudriavzevii] | KGK34314.1 |  | UDP-Glycosyltransferase/glycogen phosphorylase | Sterol 3-beta-glucosyltransferase UGT80A2 (3.40.50.2000/FF/9) | 1.000083 |
| hypothetical protein JL09_g6538 [Pichia kudriavzevii] | KGK34315.1 |  | UDP-Glycosyltransferase/glycogen phosphorylase | Sterol 3-beta-glucosyltransferase UGT80A2 (3.40.50.2000/FF/9) | 1.000042 |
| ATG26 [Candida oxycetoniae] | XP_049179558.1 | hexosyltransferase activity (GO:0016758) UDP-glycosyltransferase activity (GO:0008194) | UDP-Glycosyltransferase/glycogen phosphorylase | Sterol 3-beta-glucosyltransferase (3.40.50.2000/FF/29) | 1.000065 |
| ATG26 [Candida pseudojiufengensis] | XP_051616884.1 | hexosyltransferase activity (GO:0016758) UDP-glycosyltransferase activity (GO:0008194) | UDP-Glycosyltransferase/glycogen phosphorylase | Sterol 3-beta-glucosyltransferase (3.40.50.2000/FF/29) | 1.000018 |
| ATG26 [Candida jiufengensis] | XP_051617871.1 | UDP-glycosyltransferase activity (GO:0008194) hexosyltransferase activity (GO:0016758) | UDP-Glycosyltransferase/glycogen phosphorylase | Sterol 3-beta-glucosyltransferase (3.40.50.2000/FF/29) | 1.000035 |
| Ugt51c1p [Candida albicans SC5314] | XP_718281.2 | UDP-glycosyltransferase activity (GO:0008194) hexosyltransferase activity (GO:0016758) | UDP-Glycosyltransferase/glycogen phosphorylase | Sterol 3-beta-glucosyltransferase (3.40.50.2000/FF/29) | 1.000035 |
| hypothetical protein CAWG_01562 [Candida albicans WO-1] | EEQ43328.1 | UDP-glycosyltransferase activity (GO:0008194) hexosyltransferase activity (GO:0016758) | UDP-Glycosyltransferase/glycogen phosphorylase | Sterol 3-beta-glucosyltransferase (3.40.50.2000/FF/29) | 1.000035 |
| hypothetical protein G210_0480 [Candida maltosa Xu316] | EMG48878.1 | UDP-glycosyltransferase activity (GO:0008194) hexosyltransferase activity (GO:0016758) | UDP-Glycosyltransferase/glycogen phosphorylase | Sterol 3-beta-glucosyltransferase (3.40.50.2000/FF/29) | 1.000057 |
| hypothetical protein CTRG_00900 [Candida tropicalis MYA-3404] | XP_002546118.1 | hexosyltransferase activity (GO:0016758) UDP-glycosyltransferase activity (GO:0008194) | UDP-Glycosyltransferase/glycogen phosphorylase | Sterol 3-beta-glucosyltransferase (3.40.50.2000/FF/29) | 1.000028 |
| ATG26 [Candida metapsilosis] | KAG5420915.1 | UDP-glycosyltransferase activity (GO:0008194) hexosyltransferase activity (GO:0016758) | UDP-Glycosyltransferase/glycogen phosphorylase | Sterol 3-beta-glucosyltransferase (3.40.50.2000/FF/29) | 1.000067 |
| ATG26 [Candida theae] | XP_051606280.1 | hexosyltransferase activity (GO:0016758) UDP-glycosyltransferase activity (GO:0008194) | UDP-Glycosyltransferase/glycogen phosphorylase | Sterol 3-beta-glucosyltransferase (3.40.50.2000/FF/29) | 1.000032 |
| ATG26 [Candida margitis] | XP_051672733.1 | UDP-glycosyltransferase activity (GO:0008194) hexosyltransferase activity (GO:0016758) | UDP-Glycosyltransferase/glycogen phosphorylase | Sterol 3-beta-glucosyltransferase (3.40.50.2000/FF/29) | 1.000059 |
| uncharacterized protein CPAR2_801340 [Candida parapsilosis] | XP_036664757.1 | UDP-glycosyltransferase activity (GO:0008194) hexosyltransferase activity (GO:0016758) | UDP-Glycosyltransferase/glycogen phosphorylase | Sterol 3-beta-glucosyltransferase (3.40.50.2000/FF/29) | 1.000055 |
| unnamed protein product [Candida parapsilosis] | CAD1810499.1 | UDP-glycosyltransferase activity (GO:0008194) hexosyltransferase activity (GO:0016758) | UDP-Glycosyltransferase/glycogen phosphorylase | Sterol 3-beta-glucosyltransferase (3.40.50.2000/FF/29) | 1.000055 |

*Note that the value of signal peptide prediction above 0.9 suggests that the proteins are localized on the membrane

**Table S3** **ChemPLP fitness score from GOLD docking of all enzyme candidates with 1-dodecanol as a ligand**

| **No.** | **Candidate (Accession no.)** | **Fitness score** |
| --- | --- | --- |
| 1 | EIE90240.1 | 47.43 |
| 2 | KAG1140388.1 | 46.51 |
| 3 | KAG1302451.1 | 46.00 |
| 4 | KAG1487268.1 | 44.78 |
| 5 | KAG1493412.1 | 46.26 |
| 6 | KAG1540849.1 | 50.66 |
| 7 | KAG1135664.1 | 47.52 |
| 8 | EIE92684.1 | 46.72 |

**Supplementary Data 1 Condon optimized gene encoding EIE92684.1 candidate**

3’-ATGGCGACTGGTGAGAACACCTTCCAGCTGGCGCTGGACTACCACGTTCCGAAGAAC
ATCGTGTTCAGCTCTAGCCAGGGTGGTTCTTCTCACGTTGTTTGGGTTCTGTCCATTCTGGAAGAACTGGCGGCTCGTGGTCACCACACCATCTTCTACACTCGTGATGACCAGACCAAATTCATCAAGAACTTTCCGACCGTTGATCTGGTTTCTGCTGGTCCGGCAGTTGCGACCAAAGAAGAAATGGGTAACCTGATCCGTAACCTGACCACCAAAGAACCGGTTGATCGTCTGGTTTACATGCTGGAAGAGTTCAGCCAGAACTTCACCGAGGACTACCTGAACCACATGGACCTGTTCTCCAAGAAAGACGTTGACCTGGCTCTGTGCGGTCACATCATGACCGCGTGCGTTGAAGCGTCTATCACCAGCAAGATTCCGTTCATCTACACCTCTGTACCACTGTACTCTCTGGACACCTCTGCGCCGTACATCAACATCGACTTCTTGAGCTTCCACAACCCGTCCAACAAAGACGTTTCTTTCTTCGAACGTCTGAAAGACAAAGTTATCAATCCGATCCACTTCTTCATCAAAGCGCGTGGTTTCATCAAGAAATCTCAGCAAGAGAAGATGGCGCTGGGTTTGGAAACCACCTCTAATCCAGCGGCGAAACTGAAAGACGCGATCAAACTGTTCAACACCGCGTTCGGTTTCGCGCCGGCACGTCCGGTTGGTCCGCTGGTTGAACTGATCGGTCCGATCGTGCCAAAGACTCATCCGGTTCTGACCGACGATCTGCAGCAGTTCTTGGACTCTCACCAGAAAGTTGTTTACGTTGCGTTCGGTCAGCACGCGGTACAGGCGACCGAAGAACTGGGTCTGATCATGACCTCTATGCTGGAGAACATGGAAACCGGTGACATCGACGGTATTCTGTGGTCTGTTCGTGACATGGAACACCTGTTTCCGGAAACTCTGCAGACTCGTTCTGGTCAAATCTACCGTATCCGTGAACTGTTCGGTAAGAAAGAAGGTCAAGACATCTTGTTTCTGGAATGGGCTCCACAGGTAGCGATCTTGCACCATCCGTCTACTCGTCTGTTCTTGACTCACGGTGGTGCTGGTTCTGTTTACGAAGCTCTGTACAACGGTGTACCGATCGTTGTTTATCCATTCTTCGGTGACCAGCCGGCAGCAGCTGTTATCGCGGAAGAGAACGGTTACGGTCGTCGTATGAAGAAATCTGACCAAGAACAGGCGATCAAAGTTGTTCAAGAAGTTCTGCGTGACGATCGTTACCGTCAGAACGCGAACCGTTTCAAAGCGCTGGTTCAGATCCGTTCTAATCACGGTGTTCAGCGTGGTGCAGACGTTGTTGAAGAAGCGCTGTACCTGCACCAAGATGGTAAGATCAACCACCGTCGTGATGTGCGTCGTGACCTGAGCTTCTTGAAAGCGTACAACCTGGACCTGTACCTGTTCTCTCTGTCCGTTATCTTCGGCTCTCTGTTCGGTGTTTACCGTCTGGTTAGCTACGGCCTGAAACGTTCTTCCGTTAAAGCGAAGAAATTCCACATCTAA - 5’
